# *N*-Heterocyclic Carbene Formation in the Ionic Liquid [EMIM^+^][OAc^–^]: Elucidating Solvation Effects with Reactive Molecular Dynamics Simulations

**DOI:** 10.1021/acs.jpcb.3c02064

**Published:** 2023-06-06

**Authors:** John P. Stoppelman, Jesse G. McDaniel

**Affiliations:** School of Chemistry and Biochemistry, Georgia Institute of Technology Atlanta, Georgia 30332-0400, United States

## Abstract

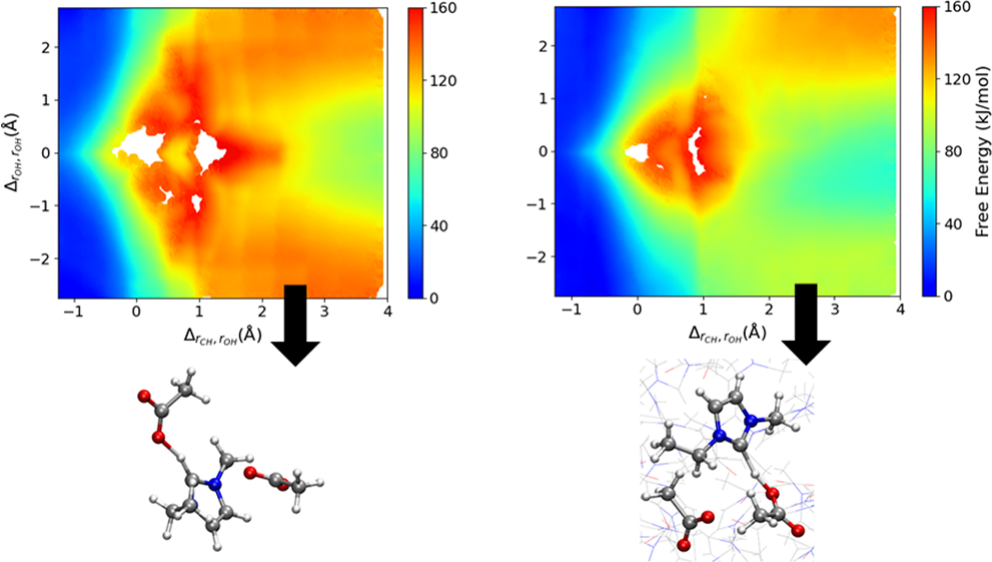

Recent experimental and theoretical work has debated whether *N*-heterocyclic carbenes (NHCs) are natively present in imidazolium-based ionic liquids (ILs) such as 1-ethyl-3-methylimidazolium acetate ([EMIM^+^][OAc^–^]) at room temperature. Because NHCs are powerful catalysts, determining their presence within imidazolium-based ILs is important, but experimental characterization is difficult due to the transient nature of the carbene species. Because the carbene formation reaction involves acid–base neutralization of two ions, ion solvation will largely dominate the reaction free energy and thus must be considered in any quantum chemical investigation of the reaction. To computationally study the NHC formation reaction, we develop physics-based, neural network reactive force fields to enable free energy calculations for the reaction in bulk [EMIM^+^][OAc^–^]. Our force field explicitly captures the formation of NHC and acetic acid by deprotonation of a EMIM^+^ molecule by acetate and in addition describes the dimerization of acetic acid and acetate. Using umbrella sampling, we compute reaction free energy profiles within the bulk IL and at the liquid/vapor interface to understand the influence of the environment on ion solvation and reaction free energies. Compared to reaction of the EMIM^+^/OAc^–^ dimer in the gas phase, the bulk environment destabilizes formation of the NHC as expected due to the large ion solvation energies. Our simulations reveal a preference for the product acetic acid to share its proton with an acetate in solution and at the interface. We predict NHC content in bulk [EMIM^+^][OAc^–^] to be on the order of parts-per-million (ppm) levels, with order-of-magnitude enhancement of NHC concentration at the liquid/vapor interface. The interfacial enhancement of NHC content is due to both poorer solvation of the ionic reactants and solvophobic stabilization of the neutral NHC molecule at the liquid/vapor interface.

## 1 Introduction

*N*-Heterocyclic carbenes (NHCs) are important catalysts in organic chemistry.^1−3^ The synthesis of the first isolable NHC by Arduengo and co-workers^4−6^ has led to an enormous number of applications in diverse fields, such as heterogeneous catalysis,^7−9^ formation of metal clusters and nanoparticles,^10−16^ development of pharmaceutical compounds,^17−19^ organocatalysis,^20−24^ and more.^1,3,25^ While a variety of methods exist for NHC synthesis, deprotonation of imidazole or imidazolium cations represents one of the most common procedures.^26,27^ In addition to being precursors to NHCs, imidazolium cations are very commonly the cationic component of room-temperature ionic liquids (ILs).^28−33^ Because ILs are utilized as both solvents in organic synthesis^34−36^ and electrolytes in electrochemical applications,^30^ the formation/presence of NHCs in such ILs would have important consequences for catalysis, reactivity, and stability.^37^ The presence of NHCs in ILs could be either beneficial (catalysis) or detrimental (electrolyte degradation), but regardless is essential to characterize for mechanistic determination.

As imidazolium cations have high p*K*_a_ values (∼22),^32^ formation of NHCs from these precursors typically requires addition of medium to strong bases,^38,39^ electrochemical reduction,^40^ and/or elevated temperatures.^41−43^ Furthermore, formation of NHCs from imidazolium cations and base must overcome the large solvation energies of the ionic reactants, the magnitude of which may depend on chemical environment. However, recent work suggests spontaneous NHC formation in imidazolium acetate ILs, despite the acetate ion being only a weak/mild base. For example, several NHC-catalyzed reactions have been found to occur in the ILs [EMIM^+^][OAc^–^] and [BMIM^+^][OAc^–^], such as benzoin condensation,^44^ formation of imidazol-2-thiones,^45^ polymerization of epoxy resins,^46^ and others, which provides indirect evidence.^47−49^ While mass spectrometry measurements show existence of NHCs in vaporized samples of [EMIM^+^][OAc^–^] and related ILs,^50,51^ the vapor phase may not be representative of the liquid due to the expected large contribution of ion solvation to the reaction free energy. No direct experimental observation of NHC formation in the liquid phase has been made, as their inherently short lifetime makes such characterization challenging. Chiarotto et al.^52^ performed cyclic voltammetry experiments and observed an oxidation peak corresponding to a NHC in [BMIM^+^][OAc^–^] at elevated temperatures (120 °C), but it was noted that the electrode itself may act as a carbene trap and thus influence the reaction.^53^ The lack of direct evidence has led to several works questioning the extent to which free carbenes exist in these systems.^53−56^ Alternative reaction pathways that do not require NHC content have been proposed for many of the aforementioned reactions, casting further doubt on whether they are spontaneously formed in these ILs at ambient conditions.^56^

Various computational/theoretical methods have been employed in order to rationalize these experimental observations. Reaction free energies calculated using implicit solvent models for both [MMIM^+^][OAc^–^] and [EMIM^+^][OAc^–^] indicate that forming the NHC is unfavorable for dielectric constant values corresponding to these ILs.^57,58^ Additionally, Gehrke et al.^54^ calculated a free energy profile of deprotonation of EMIM^+^ by acetate using *ab initio* molecular dynamics (AIMD) simulations of a 26 ion pair [EMIM^+^][OAc^–^] system. The calculated free energy (26 kcal mol^–1^) indicates that the formation of NHCs is minimal at room temperature. Additional AIMD simulations of [EMIM^+^][OAc^–^] from Brehm et al.^59^ seemed to indicate that the deprotonation of EMIM^+^ by acetate does not occur readily; however, further simulations of a free NHC in [EMIM^+^][OAc^–^] showed an interesting C···H–C bond between the carbene carbon and the EMIM^+^ methyl group that may stabilize NHCs in solution.^60^ It has been suggested that the presence of impurities or gas molecules such as CO_2_ may influence carbene formation in [EMIM^+^][OAc^–^] and similar ILs due to the possible formation of adducts.^61^

In this work, we further investigate carbene formation in the [EMIM^+^][OAc^–^] IL through reactive molecular dynamics simulations. [EMIM^+^][OAc^–^] is a common ionic liquid that has been widely studied for use in biomass processing,^62−65^ among other applications. The motivation to develop a reactive force field to study NHC formation in [EMIM^+^][OAc^–^] is based on the expected importance of ion solvation energetics on the reaction free energy and the corresponding difficulty of adequate statistical sampling of the viscous ionic liquid environment. For example, our developed approach enables much enhanced statistical sampling compared to alternative computational approaches such as *ab initio* molecular dynamics (AIMD) or hybrid quantum mechanics/molecular mechanics (QM/MM) methods.^66,67^ Our reactive force field combines physics-based expressions with neural networks and is termed “PB/NN”^68^ and constructed with an ansatz similar to the empirical valence bond (EVB) approach.^69^ In previous work,^68^ we demonstrated the ability of the PB/NN force field to capture solvation effects on the deprotonation of EMIM^+^ by acetate, but this prior work was limited to one reactive ion pair and only ion clusters (not condensed phase environments) were studied. In this work, we extend the PB/NN approach for computing NHC formation reaction free energies in the bulk [EMIM^+^][OAc^–^] ionic liquid.

Gehrke et al.^54^ have previously investigated the deprotonation of EMIM^+^ by acetate using AIMD simulations. This and studies of related systems show that there are a variety of possible proton-transfer processes, including shuttling of the proton between acetic acid and acetate molecules.^70,71^ In [EMIM^+^][OAc^–^], the acetic acid formed from the deprotonation of EMIM^+^ may share the proton with other acetate ions, with the acetic acid/acetate “dimer” being lower in free energy than a bare acetic acid molecule. To this end, our PB/NN force field is explicitly designed to model both of the following reactions:
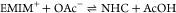
1
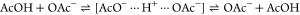
2Reaction 2 takes into account the possible sharing of the proton between acetate molecules. We note that our PB/NN force field does not explicitly model possible deprotonation of EMIM^+^ by the newly formed NHC because the purpose of the study is to investigate the initial carbene formation.

In addition to the bulk [EMIM^+^][OAc^–^] ionic liquid, we also investigate the NHC formation reaction at the ionic liquid/vapor interface. The motivation is that because ion solvation is an important contribution to the reaction free energy, the equilibria might be significantly altered (shifted toward NHC product) due to decreased solvation at the liquid/vapor interface relative to the bulk liquid. Indeed, prior work has found that a variety of reactions involving ions may have increased propensity to occur at the liquid/vapor interface,^72−74^ and the liquid/vapor interface will have pronounced importance for organic synthesis conducted in ionic liquid microdroplets.^75,76^ Whether there is enhanced formation of NHCs in [EMIM^+^][OAc^–^] at the liquid/vapor interface is of fundamental interest in light of recent reports of accelerated reactions in microdroplets and thin films^77−83^ and thus may have practical importance for applications involving this IL.

Our free energy simulations show two minima in the proton-transfer reaction profile, corresponding to the ionic reactants and the NHC product state with the proton shared as an acetic acid/acetate (AcOH/OAc^–^) dimer. Largely due to the solvation energies of the reactant ions, the computed reaction free energy is endergonic by ∼70 kJ mol^–1^ in the bulk ionic liquid. We compare the proton-transfer reaction in the bulk to the analogous reaction in the EMIM^+^/(OAc^–^)_2_ ion trimer, which is the smallest cluster enabling formation of both the NHC species and AcOH/OAc^–^ dimer with shared proton. This comparison allows elucidation of both transition state geometric effects and long-range solvation effects on the reaction free energy profile. We additionally compute the proton-transfer reaction at the [EMIM^+^][OAc^–^] liquid/vapor interface and predict order-of-magnitude enhancement of interfacial NHC resulting from solvation modulation of the interfacial environment. After describing in detail the computational PB/NN reactive force field approach in Section 2, our simulation results are presented in Section 3.

## 2 Methods

We describe our 3 × 3 multistate PB/NN Hamiltonian utilized to simulate both reactions 1 and 2 in the bulk ionic liquid and discuss the energy partitioning of matrix elements. We then briefly discuss functional forms and the parametrization approach, building off of our previous work.^68^ Finally, we detail the algorithms and software implementation utilized for our reactive molecular dynamics simulations and free energy sampling.

### 2.1 Multistate PB/NN Hamiltonian

**Figure 1:**
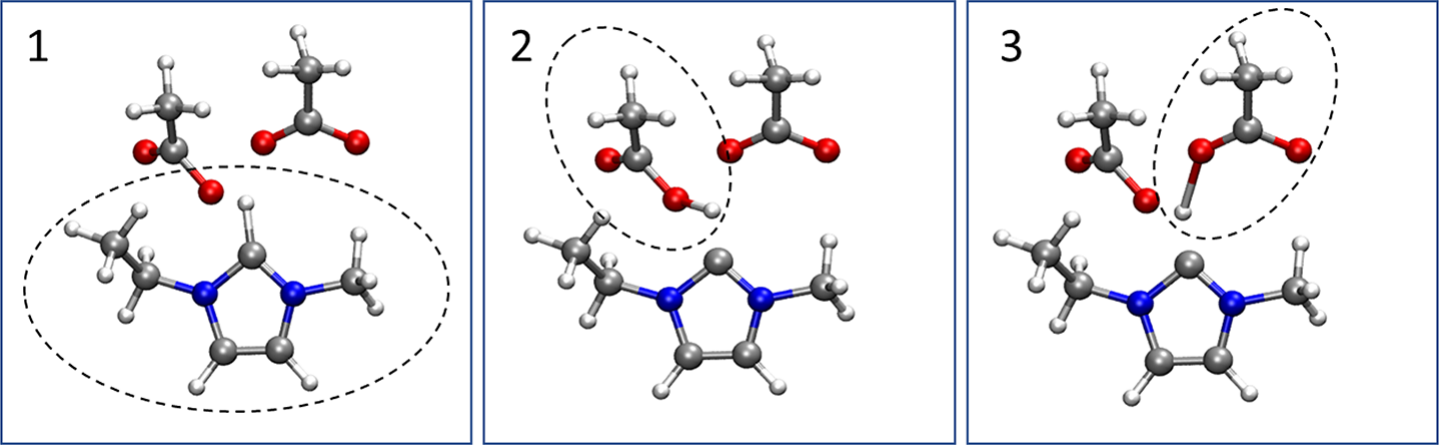
Depiction of the chemical bond topology for the three diabatic states of the reacting complex. The dashed ovals depict the molecule associated with the reacting proton in each diabatic state.

We utilize a multistate Hamiltonian in order to simulate both reactions 1 and 2 in the condensed phase; note that similar multistate Hamiltonians have been constructed in other EVB-like force fields for reactive molecular dynamics simulations.^84−94^ Our multistate PB/NN Hamiltonian used here is of 3 × 3 form, with matrix elements composed of both physics-based terms and neural networks as will be discussed:
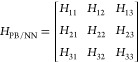
3Following the EVB ansatz,^69,87,95,96^ the diagonal terms correspond to diabatic states that have direct chemical interpretation as reactant and product species. In this case, the three different diabatic states correspond to different chemical bond topologies involving the single acidic/reactive proton, as schematically depicted in Figure 1. The off-diagonal elements couple the diabatic states to mediate the chemical reaction(s), in this case proton transfer, between the reactant(s) and product(s). Note that in the ionic liquid [EMIM^+^][OAc^–^], while every EMIM^+^ cation has an acidic proton that in principle could react, only a single tagged EMIM^+^ cation is considered “reactive” in the simulation, along with its two closest acetate ions, and this choice defines the reacting complex and diabatic states (Figure 1).

Our parametrization procedure for the multistate Hamiltonian utilizing both physics-based and neural network terms has been described in our prior work.^68,97^ The reader is referred to the previous publication for more in-depth discussion,^68^ while we summarize the key aspects of the methodology here. The diagonal elements of the Hamiltonian *H*_*ii*_ are represented by the following energy partitioning:
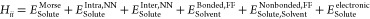
4In eq 4, each energy term is labeled according to whether it applies to the reacting complex (depicted in Figure 1 and termed the “Solute”) or to nonreacting solvent molecules (labeled as “Solvent”). Equation 4 employs both physics-based terms and neural networks. The physics-based terms are the following: *E*_Solute_^Morse^ is a Morse potential modeling bonds involving the reactive proton (which can break and form); *E*_Solvent_^Bonded,FF^ are standard harmonic bond, angle, and dihedral terms^98,99^ describing intramolecular flexibility of solvent molecules; and *E*_Solute,Solvent_^Nonbonded,FF^ encompasses physics-based, nonbonded interactions between solute/solute, solute/solvent, and solvent/solvent molecules. While in principle *E*_Solute,Solvent_^Nonbonded,FF^ could consist of generic Coulomb plus Lennard-Jones terms using parameters from standard force fields, in our implementation we utilize the SAPT-FF force field^100,101^ parametrized on the basis of symmetry-adapted perturbation theory (SAPT), so as to be consistent with our *ab initio* diabatization scheme (*vide infra*). The constant term *E*_Solute_^electronic^ is the electronic energy of the noninteracting solute molecules in the reacting complex, for the particular chemical bond topology of the diabatic state (and minimum-energy molecular geometries).

The two remaining terms in eq 4, *E*_Solute_^Intra,NN^ and *E*_Solute_^Inter,NN^, are described by neural networks. *E*_Solute_^Intra,NN^ is composed of energetics from monomer-based neural networks, one for each molecule in the reacting complex, accounting for the intramolecular vibrational energetics. These neural networks replace bonded force field terms for these molecules (e.g., bond/angle/dihedral potentials), the motivation being that the latter analytic expressions are generally not flexible enough to describe intramolecular energetics with chemical accuracy, particularly near a transition state and far from the equilibrium monomer geometries. Note that *E*_Solute_^Intra,NN^ describes all *nondissociative*, *intramolecular vibrations*, while bonds involving the reactive proton are described by the Morse potential, *E*_Solute_^Morse^ (the motivation for this partitioning is to remedy asymptotic instabilities of the neural networks, as discussed in our previous work^68^). The final term *E*_Solute_^Inter,NN^ is composed of energetics from short-range, dimer-based neural networks, which account for breakdown of the physics-based interaction terms *E*_Solute,Solvent_^Nonbonded,FF^ at very close monomer/monomer distances. Note that the diabatic decomposition of the Hamiltonian (eq 3) inherently leads to inaccurate intermolecular interactions at unphysically close distances for one or more of the diabats, for which a standard force field will exhibit uncontrolled behavior/divergence. *E*_Solute_^Inter,NN^ is meant to correct this divergent behavior, without modifying long-range physics-based interactions (e.g., electrostatics, induction, and dispersion) and is discussed in detail in our previous work.^68^

The off-diagonal terms of the Hamiltonian (eq 3), *H*_*ij*_, are symmetric (*H*_*ij*_ = *H*_*ji*_) and describe the coupling between diabatic states along the reaction coordinate. While prior EVB approaches typically have employed analytic functional forms for these off-diagonal elements,^69,102^ we choose to utilize neural networks for the *H*_*ij*_ to provide a more versatile description of the diabatic coupling and enable accurate rendering of the adiabatic potential energy surface (PES) for general dimensionality. The neural networks utilized for *H*_*ij*_ depend on coordinates of the dimer (acid/base) for which the bond topology changes between diabats *i* and *j*. Note that there is no dependence of *H*_*ij*_ on the solvent; this is in line with the ansatz of previous EVB approaches^69,103^ and means that solvent interactions with the reacting complex are only explicitly incorporated into the diagonal terms. The success of this ansatz for capturing solvent modulation of reaction free energies has been previously demonstrated by others^69,95^ as well as in our recent work.^68^

### 2.2 PB/NN Parameterization and Neural Network Architecture

The terms *E*_Solute_^Morse^, *E*_Solute_^Intra,NN^, *E*_Solvent_^Bonded,FF^, *E*_Solute,Solvent_^Nonbonded,FF^, and *E*_Solute_^electronic^ have been parametrized in our previous work^68^ and will only be briefly discussed. The monomer neural networks constituting *E*_Solute_^Intra,NN^ utilize the SchNet architecture^104^ and are trained to AIMD generated data at the PBE-D3(BJ)/aug-cc-pVTZ level of theory, as consistent with our prior work.^68^ We note that prior studies have benchmarked DFT functionals for their ability to accurately model the electronic structure and reactivity of various carbene species.^105−107^ In this work, we have refined the original parametrization of *E*_Solute_^Intra,NN^ terms to better capture the *anti* configuration of acetic acid in the condensed phase (see the Supporting Information). *E*_Solvent_^Bonded,FF^ handles all bonded terms within the solvent. The EMIM^+^ parameters are taken from prior work,^108^ and the acetate parameters were fit using the ForceBalance package.^109^ These force field parameters can be found within the Supporting Information. *E*_Solute,Solvent_^Nonbonded,FF^ handles nonbonded intramolecular interactions in solvent molecules, all intermolecular solvent–solvent and solvent–solute interactions, and all asymptotic intermolecular interactions between solute molecules. We use the SAPT-FF force field for this term,^100^ as parametrized previously.^68^ The final term in the diagonal Hamiltonian elements (eq 4) that needs to be parametrized is *E*_Solute_^Inter,NN^; as mentioned, this is effectively a correction to the intermolecular interaction at small separation distances. We utilize the AP-Net architecture for these neural networks, as AP-Net has been explicitly designed for modeling intermolecular interactions.^110^*E*_Solute_^Inter,NN^ is then trained to the difference between SAPT0 *ab initio* data^111^ and the *E*_Solute,Solvent_^Nonbonded,FF^ energy (SAPT-FF) for dimer configurations. For this fitting, the so-called “delta Hartree Fock” term is not included, so that the energy is nonvariational and purely based on intermolecular perturbation theory. Thus, the “diabatization” defining the diagonal elements (eq 4) is based on perturbation theory, and the pros/cons of this approach are discussed in our previous work,^68^ with elaboration provided in the Supporting Information. In this work, we develop new parametrization for the acetic acid/acetate dimer interaction in *E*_Solute_^Inter,NN^ with comprehensive details given in the Supporting Information. *E*_Solute_^electronic^ is the gas-phase energy of the isolated monomers within each diabat as computed previously at the PBE-D3(BJ)/aug-cc-pVTZ level of theory.^68^

**Figure 2:**
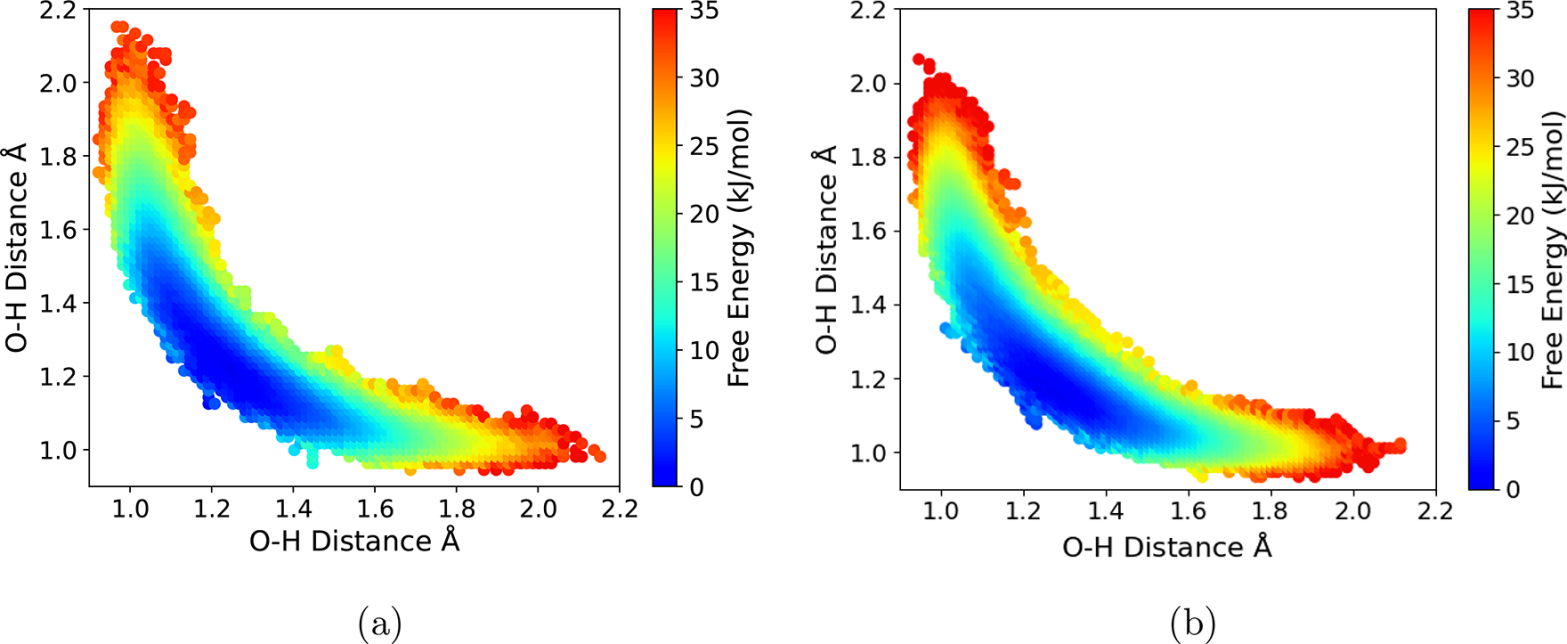
Free energy surfaces for the proton-transfer reaction between acetic acid and acetate ion, as computed from (a) AIMD and (b) PB/NN simulations. The free energy is plotted as a function of the distances between the acidic proton and closest oxygen atoms of the two acetate ions.

For the off-diagonal elements of the Hamiltonian (eq 3), we utilize neural networks consisting of a modified AP-Net architecture.^68,110^ For this work, the specific off-diagonal element *H*_*ij*_ mediating the acetic acid/acetate proton transfer was trained (i.e., coupling the diabats involving acetic acid bond topologies), while the other off-diagonal elements were based upon previous work.^68^ For training, PBE-D3(BJ)/aug-cc-pVTZ energies and forces for the (gas-phase) acetic acid/acetate dimer were generated along the (adiabatic) proton-transfer reaction coordinate. The off-diagonal *H*_*ij*_ element is fit through minimization of the appropriate 2 × 2 subblock of the Hamiltonian (eq 3) to the *ab initio* data for the adiabatic (gas-phase) reaction surface. Details of the *ab initio* data generation, neural network architecture and parameters, and fitting approach are given in the Supporting Information. Figure S2 shows the final force field fit to the DFT PES, with an MAE of 0.81 kJ mol^–1^ for the total test set. As a further test for the fidelity of modeling the acetic acid/acetate proton-transfer reaction, we computed reaction free energy surfaces using umbrella sampling, with both AIMD and the PB/NN reactive force field. Computational details are given in the Supporting Information. The resulting free energy surfaces are shown in Figure 2, with the trained PB/NN predictions in excellent agreement with AIMD.

An important aspect of the method is that for each neural network involved in modeling the atoms in a dissociating bond (this includes *E*_Solute_^Intra,NN^ neural networks for EMIM^+^ and acetic acid, the *E*_Solute_^Inter,NN^ and *H*_*ij*_ neural networks), the output is multiplied by a Fermi–Dirac damping function (eqs S1 and S5) that takes as input the value of the respective dissociating bond distance. For the off-diagonal terms, *H*_*ij*_, the neural network(s) are multiplied by two Fermi–Dirac functions, as there are two possible dissociating bonds for the reactant and product. As neural networks are incapable of extrapolation outside of the training set, the damping functions restrict each neural network output to a well-defined region of phase space, preventing uncontrolled predictions. The specific details of the damping functions have been slightly modified from our previous work,^68^ as discussed in the Supporting Information.

We finally note the pros/cons of our choices for functional form of the Hamiltonian matrix elements in eq 3. Our PB/NN model incorporates both a finer degree of energy partitioning and more complex terms (e.g., neural networks) compared to more standard EVB models,^69^ which makes parametrization more complex/tedious and requires a large number of *ab initio* calculations (*vide infra*). The benefit of the added complexity/neural networks is the versatile, high-accuracy rendering of both diabatic and adiabatic PES, in general dimensionality, relative to the underlying *ab initio* PES. This enables direct application of the Hamiltonian (eq 3) for molecular dynamics simulation in the condensed phase. For example, in both this and previous work,^68^ we show that our PB/NN approach produces an adiabatic PES with chemical accuracy relative to the underlying density functional theory (DFT), *ab initio* description, and enables prediction of reaction free energies in different chemical environments in quantitative agreement with AIMD simulations.^68^ We include timings for the various terms in the PB/NN Hamiltonian in the Supporting Information.

### 2.3 Reactive Molecular Dynamics Simulations

Utilizing our multistate PB/NN Hamiltonian (eq 3), reactive molecular dynamics simulations are conducted to compute reaction free energies in bulk [EMIM^+^][OAc^–^] and at its liquid/vapor interface. We utilize a hybrid software implementation, with both OpenMM^112^ and PyTorch^113^ libraries used to calculate energies/gradients of the different Hamiltonian matrix elements (eq 3) as defined by their energy partitioning (eq 4). The adiabatic atomistic forces are obtained from the ground state eigenvector of eq 3 and derivatives of each Hamiltonian matrix element, based on the Hellmann–Feynman theorem.^68^ The ASE calculator is utilized to run the MD simulations,^68,114^ with Plumed^115^ utilized for umbrella sampling simulations. Note that all workflow is wrapped in Python, employing the Python APIs of these software libraries. Our implementation can be found on our github site and in the Supporting Information.^116^

Simulations were run for three different systems/environments. The first system is the isolated reaction complex, consisting of one EMIM^+^ cation and two acetate anions in the gas phase without periodic boundary conditions. The second system contains 40 EMIM^+^/acetate ion pairs and is designed to represent the bulk liquid. Periodic boundary conditions were used, with cubic box dimensions of 22.08 Å for each side. One EMIM^+^ cation is randomly selected to be “reactive”, with the two closest acetate anions defining the reacting complex (Figure 1). The third system that we simulate is the [EMIM^+^][OAc^–^] vapor/liquid interface. A vacuum gap is added to the previous bulk system, creating a new simulation box with dimensions of 22.08 Å × 22.08 Å × 66.24 Å. For the latter vapor/liquid interface system, we utilize a standard 3D Ewald/PME treatment of electrostatics, without slab correction.^117^ This is because the slab system is symmetric, with no net average dipole moment (and vacuum gap of ∼44 Å) for which any such slab correction is expected to be insignificant.^117,118^

The reacting complex consists of one EMIM^+^ and two acetate anions. We use umbrella sampling with MD simulations in order to calculate free energy profiles encompassing the two different proton-transfer processes shown in reactions 1 and 2. The collective variables utilized for umbrella sampling are
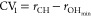
5a
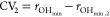
5bThe CVs are differences of distances: The first CV (eq 5a) is the difference between the EMIM^+^ carbon–reactive proton distance (*r*_CH_) and the shortest acetate oxygen–reactive proton distance . The second CV (eq 5b) is the difference between the shortest acetate oxygen–reactive proton distance  and the second shortest acetate oxygen–reactive proton distance . All acetate oxygens are considered in the *r*_OH_ values computed in eq 5, with the condition that the O atom in  must be on a different molecule than the O atom in . The reaction (different protonation states of all three molecules) is then sampled by applying umbrella potentials in the 2D space spanned by these CVs. Each umbrella potential  uses a 200 kJ/(mol Å^2^) force constant. For all systems/environments, umbrellas were applied spanning values for CV_1_ ranging from −1.0 to 3.5 Å, increasing in increments of 0.5 Å. The value of CV_2_ ranged from −2.5 to 2.5 Å, increasing in increments of 0.25–0.3 Å. This results in approximately ∼200 windows per umbrella sampling simulation, and the WHAM procedure is subsequently used to construct 2D PMFs from each set of umbrella sampling simulations.^119^ Each window is simulated for 40 ps in the NVT ensemble at 300 K, controlled by a Langevin thermostat with friction coefficient of 1 ps^–1^. The simulation time step is set to 0.5 fs for all simulations. For the liquid and liquid/vapor interface simulations, particle mesh Ewald (PME) is used for long-range electrostatics,^120^ and van der Waals interactions are truncated/cut off at 1.1 nm, as necessitated by the system box sizes. Drude oscillators are utilized to model polarization within the *E*_Solute,Solvent_^Nonbonded,FF^ terms, with Drude oscillator positions (adiabatically) optimized using the “DrudeSCFIntegrator” in OpenMM.^112^ An anharmonic restraining potential is utilized to prevent Drude oscillator divergence at short contact distance, as described by Huang et al.^121^ For the liquid/vapor interface simulations, we apply a restraint of approximately 20 kJ mol^–1^ to the center of mass of the reacting complex, centered at the Gibbs dividing surface (Figure S9).

We finally discuss practical issues that inherently arise from the creation of a “reacting complex” or “active zone” in the liquid simulations. In our case, the “reacting complex” is defined as the specified EMIM^+^ ion and its two closest acetate anions. During the simulation, it is of course possible for this complex to change identity if a solvent acetate ion displaces one of the two closer acetate ions in the EMIM^+^ coordination, so that the reacting complex must be redefined as a function of simulation time. This is analogous to similar issues dealt with in QM/MM simulations of solution-phase chemical reactions, for which “adaptive” QM/MM schemes have been developed.^122−125^ In adaptive QM/MM schemes, the goal is to switch between quantum mechanical and molecular mechanics descriptions for molecules moving in/out of the active zone, while minimizing introduction of spurious forces. Because definition of an active zone is usually distance based, discontinuities will arise at the boundary due to application of different Hamiltonian terms in the inner and outer regions. If a smoothing or switching function is introduced as an attempted remedy, then a spurious force term will arise from the switching function.

We describe how we deal with the issue of redefining the reacting complex as a function of simulation time for our PB/NN model. If the identity of acetate ions changes in the reacting complex (e.g., from a solvent acetate ion displacing a closer coordination acetate ion), the PB/NN Hamiltonian is “reinitialized” to the new reacting complex identity. As discussed below, each “reinitialized” term will have different effects on the energy conservation for the PB/NN Hamiltonian. It is important to note that during such “reinitialization”, the PB/NN Hamiltonian (eq 3) has essentially collapsed to a 2 × 2 matrix, in terms of its description of the adiabatic ground state. This is because the acetate ions that switch in/out of the reacting complex are sufficiently far away from the acidic proton that the corresponding protonated diabatic state is very high in energy (due to the Morse potential) relative to the other diabatic states. This minimizes adiabatic force discontinuities, as discontinuities in a (high energy) diabat may not propagate to the adiabatic forces. For example, switching the Morse potential (with a harmonic bond) and off-diagonal coupling *H*_*ij*_ applied to different acetate ions during “reinitialization” should have little-to-no effect on the adiabatic forces due to the mentioned rationale.

Force discontinuities will, however, arise from the intramolecular energetic terms of the acetate ions. This is because the acetate ions in the reacting complex are modeled with a neural network (*E*_Solute_^Intra,NN^), while solvent acetate ions are modeled with a standard force field (*E*_Solvent_^Bonded,FF^) for these energetics. There will thus be a jump/discontinuity in energy/forces when acetate ions are switched between these models, an issue that cannot be straightforwardly corrected with a smoothing/switching function (which would introduce spurious forces). We thus apply the neural network description for intramolecular energetics (*E*_Solute_^Intra,NN^) to a broader set of acetate ions (specifically the six closest acetate ions to the acidic proton) than just the two acetate ions in the reacting complex. When one of the acetate ions previously considered a solvent molecule moves into the reacting complex, this term thus does not need to be reinitialized. We find that applying the *E*_Solute_^Intra,NN^ term to the six acetate molecules closest to the reacting EMIM^+^ is sufficient for ensuring no acetate molecule with the *E*_Solvent_^Bonded,FF^ intramolecular energy description diffuses into the reacting complex during the time scale of our simulations.

More details for the “reinitialization” of the reacting complex, and subsequent effects on energy conservation, are discussed in the Supporting Information (note there is an effect from the *E*_Solute_^Inter,NN^ terms, as discussed in the Supporting Information). Energy conservation is the rigorous test for a conservative force field implementation (i.e., no cutoffs, jumps/discontinuities, and drift), and we thus we run PB/NN simulations in the NVE ensemble to benchmark the above treatment/approximations. Energy conservation benchmarks are shown in Figures S3–S5. We do observe energy jumps of order ∼10 kJ mol^–1^, which we note are most likely due to switching the *E*_Solute_^Inter,NN^ terms. In this regard, there are two important points to note: First, the magnitude of these jumps is much smaller than typical energy fluctuations caused by an NVT thermostat. Second, the total energy drift is comparable to that of a baseline (nonreactive) OpenMM simulation, as limited by energy drift from the Drude oscillators (imperfect convergence). For these reasons, it is expected that our simulation predictions for reaction free energies and other thermodynamic properties are largely unaffected by this issue.

## 3 Results and Discussion

In Section 3.1, we first compare the free energies for the proton transfer of EMIM^+^/OAc^–^ dimer and EMIM^+^/(OAc^–^)_2_ trimer in the gas phase and then analyze the proton-transfer reaction in the bulk [EMIM^+^][OAc^–^] ionic liquid. In Section 3.2, we analyze the proton-transfer reaction occurring at the [EMIM^+^][OAc^–^] ionic liquid/vapor interface and compare/contrast interfacial solvation effects relative to solvation in the bulk [EMIM^+^][OAc^–^] ionic liquid.

### 3.1 Proton-Transfer Free Energies for EMIM^+^/OAc^–^, EMIM^+^/(OAc^–^)_2_, and Bulk [EMIM^+^][OAc^–^] Ionic Liquid

**Figure 3:**
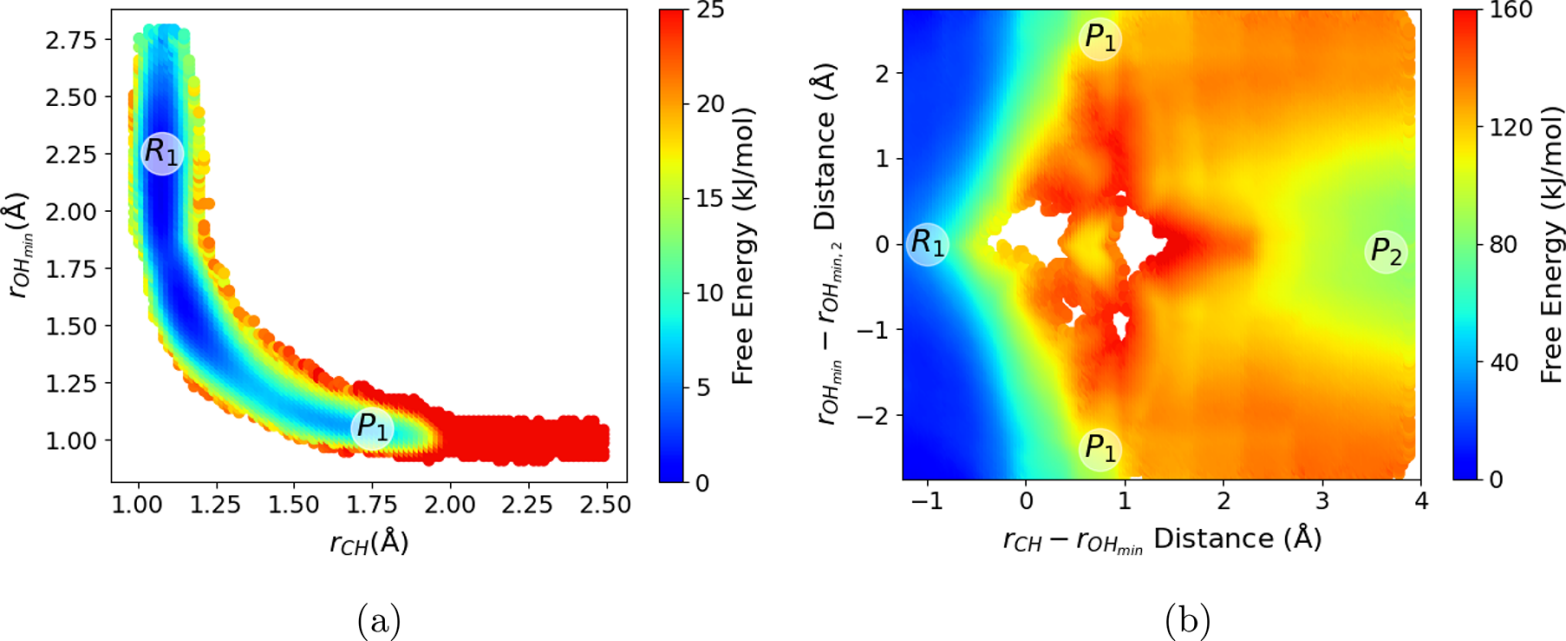
(a) Proton-transfer free energy for EMIM^+^/OAc^–^ dimer in the gas phase. (b) Proton-transfer free energy for EMIM^+^/(OAc^–^)_2_ trimer in the gas phase. The labels *R*_1_ and *P*_1_ correspond to the reactants and products of eq 1. The *P*_2_ label corresponds to the shared proton between two OAc^–^ molecules in eq 2.

Because proton transfer between EMIM^+^ and OAc^–^ is substantially influenced by the solvation energies of the ions, it is insightful to analyze how the proton-transfer free energy changes from gas-phase ion clusters (dimers and trimers) to the bulk ionic liquid. We have previously computed the proton-transfer free energy for the EMIM^+^/OAc^–^ dimer in the gas phase.^68^ Here we compute the corresponding reaction free energy for the EMIM^+^/(OAc^–^)_2_ trimer (using the extended 3 × 3 Hamiltonian, eq 3) and compare the dimer/trimer reaction free energies in Figure 3. The umbrella sampling procedure utilized for free energy calculations is the same as in our prior work and is detailed in the Supporting Information.

Figure 3a shows the proton-transfer free energy for EMIM^+^/OAc^–^ dimer, as a function of CVs “*r*_CH_” and “”. Figure 3b shows the proton-transfer free energy for the EMIM^+^/(OAc^–^)_2_ trimer as a function of the CVs defined in eq 5. The “*R*_1_” and “*P*_1_” labels in both Figures 3a and 3b correspond to the points of the profile in which the reactant and product in eq 1 are formed, while the “*P*_2_” label in Figure 3b corresponds to the AcOH/OAc^–^ dimer with shared proton configuration (eq 2). It is important to note that the free energy scales of Figures 3a and 3b are very different; the proton transfer for the EMIM^+^/OAc^–^ dimer is relatively isoenergetic, so that Figure 3a is plotted with a narrow free energy range (∼25 kJ mol^–1^). In contrast, the additional ion in the EMIM^+^/(OAc^–^)_2_ trimer complex modulates the proton transfer such that the equilibrium is substantially shifted toward ionic reactants, and correspondingly the free energy profile in Figure 3b encompasses a much larger range (∼160 kJ mol^–1^).

For the gas-phase EMIM^+^/OAc^–^ dimer, proton transfer to form the carbene species occurs rapidly, on a thermal energy scale (≤10 kJ mol^–1^). Figure 3a shows a broad low-energy basin for *R*_1_ (EMIM^+^/OAc^–^) at short *r*_CH_ distances (∼1.1 Å) and longer *r*_OH,min_ distances (≥1.25 Å). The formation of *P*_1_ (NHC/AcOH) at *r*_CH_ = 1.75 Å and *r*_OH, min_ = 1.0 Å is essentially a barrierless reaction for the EMIM^+^/OAc^–^ dimer, with a small free energy increase of ∼10 kJ mol^–1^. At this point, there is a stabilizing O–H···C hydrogen bond between the formed NHC and acetic acid; there is an accompanied increase in free energy as the *r*_CH_ distance increases, and this hydrogen bond is broken. Rapid proton transfer converts the EMIM^+^/OAc^–^ dimer between the two different protonation state topologies on approximately picosecond time scales, as observed previously in AIMD simulations.^68^

In contrast, proton transfer to form the carbene species incurs a significantly higher free energy cost for the EMIM^+^/(OAc^–^)_2_ gas-phase trimer, as shown in Figure 3b. As will be discussed, this is due to the “solvation” energy of the ionic reactants due to the additional acetate anion in the EMIM^+^/(OAc^–^)_2_ complex. The reaction in eq 1 can proceed with either acetate in the reacting complex, so we show two labels that correspond to *P*_1_. A value of 0 on the *y*-axis of Figure 3b indicates that the proton is equidistant to the acceptor oxygen on both acetate molecules, while positive or negative values indicate that one acetate molecule is closer to the proton than the other. Similar to the dimer system, the trimer global free energy minimum corresponds to the ionic reactants (i.e., Figure 1, panel 1). As the minimum free energy state is located at *x*-axis values of ∼−1 Å and a broad range of values along the *y*-axis, there appears to be little preference for the specific orientation of the acetates with respect to the EMIM^+^ ring; rather, the preference is for each species in the reacting complex to remain ionic, with substantial electrostatic stabilization. The minimum free energy pathway from *R*_1_ to *P*_1_ proceeds along *y*-axis values ≥2 and ≤−2 in Figure 3b, indicating reaction 1 is further inhibited if the nonreacting acetate is too close to the EMIM^+^ and OAc^–^ engaged in the deprotonation reaction. The favorable electrostatic interactions with the “spectator” acetate anion leads to a free energy cost of ∼90 kJ mol^–1^ to form the neutral products (*P*_1_) from the ions, which is substantially larger than for the gas-phase dimer.

Figure 3b shows that the proton transfer within the EMIM^+^/(OAc^–^)_2_ trimer proceeds through a transition state, before reaching the local free energy minimum at *P*_2_ corresponding to the NHC species and AcOH/OAc^–^ dimer with shared proton. The free energy of this NHC “product” state (*P*_2_) is ∼70–80 kJ mol^–1^ higher than the EMIM^+^/(OAc^–^)_2_ ionic reactant state. The transition state occurs in a region between *P*_1_ and *P*_2_ with a configuration depicted by simulation snapshots in Figure S12. At the transition state, the nonreacting “spectator” acetate is oriented above the plane of the imidazolium ring and hydrogen bonded to one of the ring nonreactive protons, as the formed NHC/AcOH engage in hydrogen bonding (Figure S12c,d). The transition state between *P*_1_ and *P*_2_ has a relative free energy of 130 kJ mol^–1^, which is caused by unfavorable geometries initiating the formation of the AcOH/OAc^–^ dimer close to the NHC ring. The local free energy minimum at *P*_2_ corresponding to the NHC species and AcOH/OAc^–^ dimer product only occurs when the distance between the AcOH/OAc^–^ dimer is sufficiently far away from the NHC ring.

There are two major takeaways from comparing the gas-phase, proton-transfer reactions for the EMIM^+^/OAc^–^ dimer and EMIM^+^/(OAc^–^)_2_ trimer. First, there is a clear influence of “solvation” energy in stabilizing the ionic reactants (EMIM^+^ and OAc^–^ species) relative to carbene product (NHC and AcOH species). With the additional “spectator” OAc^–^ anion in the EMIM^+^/(OAc^–^)_2_ trimer, the NHC products “*P*_1_” are shifted roughly ∼80 kJ mol^–1^ higher in free energy, relative to the similar reaction coordinate for the EMIM^+^/OAc^–^ dimer. This is because the spectator OAc^–^ anion “solvates” the ionic EMIM^+^/OAc^–^ reactants, substantially lowering the free energy of the ionic state (*R*_1_). There is no such solvation for the gas-phase EMIM^+^/OAc^–^ dimer, which is the reason the ionic/neutral (reactant/product) states are nearly isoenergetic for (only) the dimer complex. As we will show, the proton-transfer reaction profile in the [EMIM^+^][OAc^–^] bulk ionic liquid is qualitatively similar to that of the EMIM^+^/(OAc^–^)_2_ trimer; this is interesting, as it indicates that the single “spectator” OAc^–^ anion captures a “large chunk” of the actual solvation energy in the liquid. The second major takeaway from Figure 3b is the importance of the shared proton, AcOH/OAc^–^ dimer configuration. When the NHC species is formed, the stable proton state is the AcOH/OAc^–^ dimer complex and not a bare AcOH molecule; the importance of this bonded topology was the motivation for the 3 × 3 Hamiltonian developed for the simulations. Thus, the solvation energy of the AcOH/OAc^–^ dimer is an important contributor to the overall reaction free energy for NHC formation, as will be analyzed in more detail for the bulk [EMIM^+^][OAc^–^] ionic liquid.

**Figure 4:**
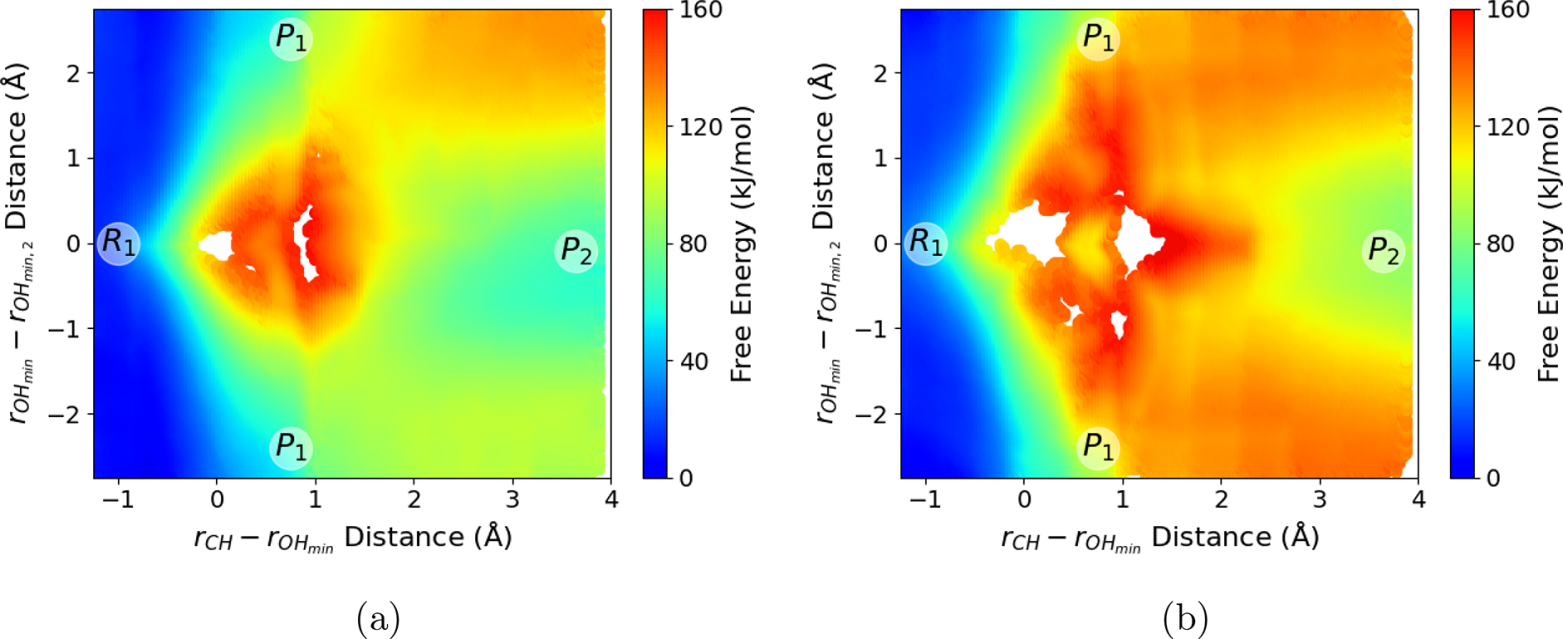
(a) Proton-transfer free energy within the bulk [EMIM^+^][OAc^–^] ionic liquid. (b) Proton-transfer free energy for EMIM^+^/(OAc^–^)_2_ trimer in the gas phase.

We next discuss our simulations of the proton-transfer reaction in the bulk [EMIM^+^][OAc^–^] ionic liquid. As discussed above, the EMIM^+^/(OAc^–^)_2_ trimer is the better reference system to provide context for proton transfer in the [EMIM^+^][OAc^–^] ionic liquid, and thus we directly compare reaction free energies for these systems. In Figure 4 we show the reaction free energy profile computed for the [EMIM^+^][OAc^–^] ionic liquid and compared to the previous profile computed for the gas-phase, EMIM^+^/(OAc^–^)_2_ trimer. The immediate observation is the qualitative similarity between the proton-transfer reaction profiles computed in the [EMIM^+^][OAc^–^] ionic liquid (Figure 4a) and gas-phase EMIM^+^/(OAc^–^)_2_ trimer (Figure 4b). The reaction profile in the [EMIM^+^][OAc^–^] ionic liquid shows similar free energetic trends going from the ionic reactants at *R*_1_ to the transition state region between *P*_1_ and *P*_2_, to the product state at *P*_2_ encompassing the NHC species and AcOH/OAc^–^ dimer complex. There are, however, quantitative differences between these free energy profiles resulting from the extended solvation environment of the liquid state. Both the “transition state” between *P*_1_ and *P*_2_ and the *P*_2_ product are significantly lower in free energy in the ionic liquid phase. This indicates greater stabilization of these intermediates relative to the ionic reactants (*R*_1_) in the extended ionic liquid compared to the EMIM^+^/(OAc^–^)_2_ trimer cluster.

**Figure 5:**
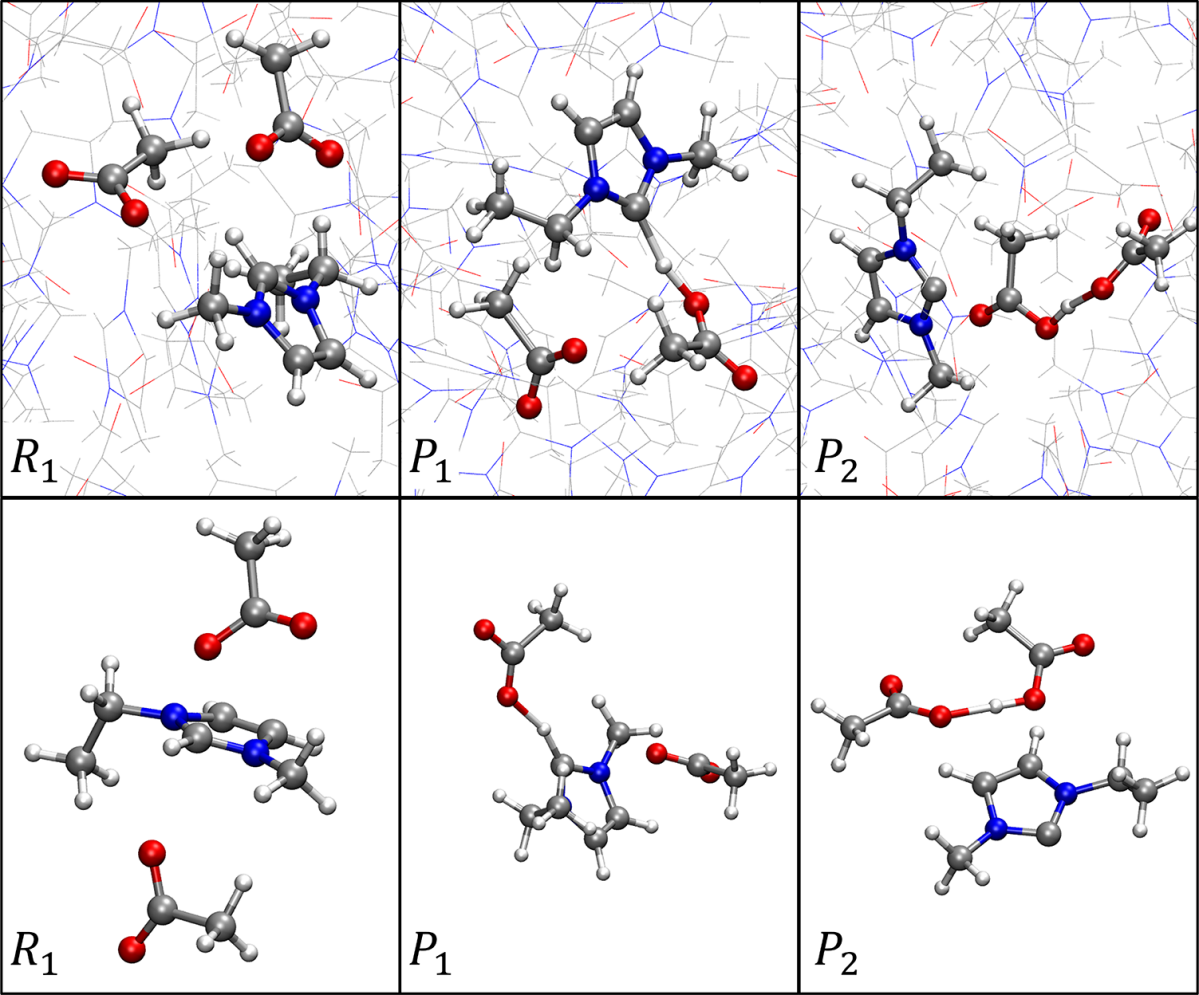
Simulation snapshots depicting reacting complex intermediates. The top panel displays snapshots from the [EMIM^+^][OAc^–^] ionic liquid simulations, and the bottom panel displays snapshots for corresponding intermediates from the gas-phase EMIM^+^/(OAc^–^)_2_ trimer simulation. The labels match the corresponding locations on the reaction free energy profiles of Figure 4.

In Figure 5, we show snapshots of the various intermediates along the reaction profile from both the [EMIM^+^][OAc^–^] ionic liquid and gas-phase EMIM^+^/(OAc^–^)_2_ trimer simulations, with more snapshots shown in Figures S11 and S12. As previously mentioned, the ionic reactants exhibit a broad free energy (global) minimum basin spanning *R*_1_, with free energy largely insensitive to complex orientation. This is demonstrated in Figures S11a,b and S12a,b, which depict visually different reacting complex configurations that are approximately isoenergetic. The free energy along the reaction path proceeding from *R*_1_ to *P*_2_ is, however, significantly different for the ionic liquid compared to the EMIM^+^/(OAc^–^)_2_ trimer cluster. This is largely due to differences in solvation and/or geometrical configuration of the key intermediates along the reaction coordinate. Figures 5 and S11–S12 show simulation snapshots depicting configurations of the reaction intermediates, within both the [EMIM^+^][OAc^–^] ionic liquid and gas-phase EMIM^+^/(OAc^–^)_2_ trimer. For the EMIM^+^/(OAc^–^)_2_ trimer, as mentioned previously, the “spectator” acetate anion hydrogen bonds to one of the protons on the EMIM^+^ ring and then orients itself above the ring plane during the course of the proton-transfer reaction. In the liquid phase, additional solvent acetate anions instead participate in this hydrogen bonding with EMIM^+^, allowing the corresponding “spectator” acetate anion to position closer to the EMIM^+^ ethyl group in the reacting complex configuration (Figure 5 “*P*_1_” panel and Figure S11c). This is a key difference, requiring breaking of a hydrogen bond to reach *P*_1_ for the gas-phase EMIM^+^/(OAc^–^)_2_ trimer, but not when the reaction proceeds in the [EMIM^+^][OAc^–^] ionic liquid. The result is that the intermediate *P*_1_ preceding the transition state is ∼35 kJ mol^–1^ lower in free energy in the liquid phase compared to the trimer cluster. This free energy difference propagates along the reaction profile toward the transition state, so that the transition state is ∼20–40 kJ mol^–1^ lower in relative free energy in the ionic liquid compared to the gas-phase EMIM^+^/(OAc^–^)_2_ trimer. Simulation snapshots depicting the transition state configuration in both the liquid and trimer cluster are shown in Figures S11d and S12d.

To more quantitatively demonstrate the differing reacting complex configurations, in Figure S14 we plot a histogram of the distance between the NHC ring center of mass and the AcOH/OAc^–^ dimer center of mass for several points along the reaction coordinate spanning *P*_1_ to *P*_2_; also shown are histograms of the angle between the NHC carbon engaged in the deprotonation reaction, the NHC ring center of mass, and the AcOH/OAc^–^ dimer center of mass, which provide information about the orientation of the dimer with respect to the imidazole plane. The distance histograms show that the dimer is farther away from the NHC center of mass in the liquid phase than the gas-phase trimer for all points along the reaction coordinate. The distribution of the angle histograms shows that the AcOH/OAc^–^ dimer is in plane with the imidazolium ring in the liquid phase (angles close to 0°) and above this plane in the gas-phase trimer (angles close to 90°). These results further indicate that the extended solvation environment distinctly modulates the reacting complex configurations. The reactive trimer is packed closer together in the gas phase, leading to higher free energies while coordinating the individual proton transfers.

Our prediction for the free energy of the initial proton transfer (reaction 1) in the [EMIM^+^][OAc^–^] ionic liquid is in semiquantitative agreement with prior AIMD studies. Gehrke et al.^54^ calculated a free energy of ∼100 kJ mol^–1^ for the deprotonation reaction (eq 1) in the liquid phase; this free energy corresponds to the transition state region of our profile slightly past *P*_1_. We compute a free energy of 90 ± 10 kJ mol^–1^ for this region of the reaction profile in the liquid phase, which is close to the prior AIMD result. We note that any asymmetry in our computed free energy profile spanning CV_2_ = 0 (along the vertical axis) is due to statistical uncertainty and is the origin of the stated uncertainty in our prediction. Such statistical uncertainty arises from the viscous nature of the ionic liquid (and correspondingly slow dynamics), making converged statistical sampling difficult.^126^ Indeed we have observed that the finite simulation time leads to different distributions of solvent molecules around the imidazolium ring for symmetric values of CV_2_, indicating somewhat incomplete sampling. We show the varying solvent orientations through spatial distribution functions (SDFs), computed with TRAVIS and plotted with ChimeraX in Figure S16.^127,128^

**Figure 6:**
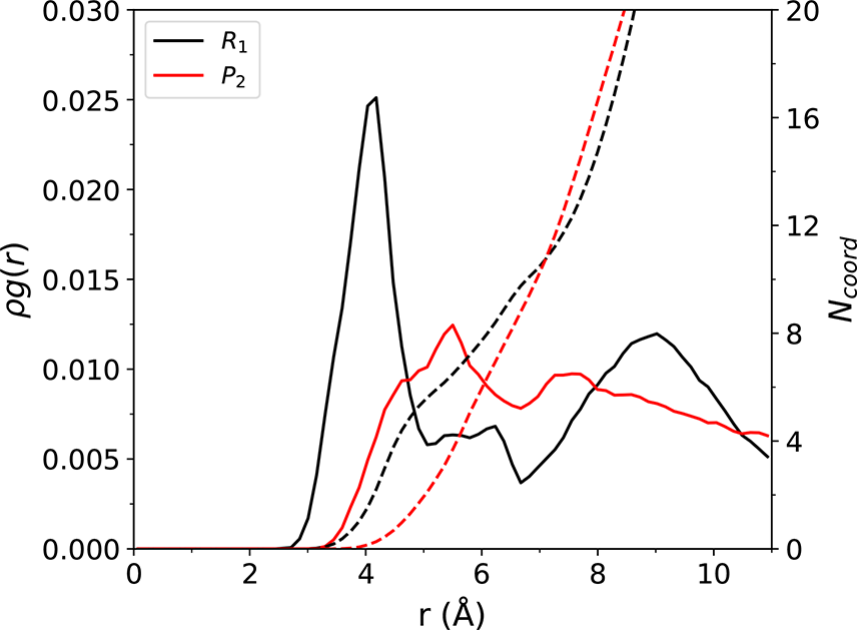
RDFs between the center of mass of the EMIM^+^ ring and acetate oxygen atoms (“*R*_1_”) and between the center of mass of the NHC ring and acetate oxygen atoms (“*P*_2_”). The corresponding coordination number at a given distance is shown by the dashed lines.

We next characterize the solvation structure around the reacting complex in the liquid phase though analysis of radial distribution functions (RDFs). In Figure 6, we plot ρ*g*(*r*) and running coordination numbers for the EMIM^+^ ring/acetate oxygen at location “*R*_1_” in Figure 4a and the NHC ring/acetate oxygen at location “*P*_2_”. These points of the profile correspond to the initial reactant and final product from our simulations. To improve statistics, these RDFs are computed from a total of 360 ps of simulation for the specific configuration along the reaction coordinate. From inspection of the RDFs, it is clear that the anion coordination surrounding the reacting complex is reduced upon formation of the neutral NHC species. In the “*R*_1_” RDF, the first peak at 4 Å corresponds to oxygen atoms present in three different acetate anions, with *N*_coord_ = 4. The first acetate is part of the reacting complex and has two oxygen atoms that are close to equidistant to the EMIM^+^ center of mass (see panel R1 in Figure 5 for an example of the configuration). The second acetate also belongs to the reacting complex but has one oxygen oriented toward the reacting proton and the other oxygen oriented toward the surrounding solvent, so that only one oxygen from this anion contributes to the peak. The third acetate is hydrogen bonded to one of the nonreactive protons and also contributes one oxygen atom to the coordination number. There is also a peak at ∼9 Å which is indicative of coordination from acetate anions in secondary solvation shell. For the NHC “*P*_2_” RDF, in contrast to EMIM^+^, the acetates are positioned farther away from the ring. There is a peak at ∼5.5 Å composed of the reacting complex oxygen atoms and various other oxygens surrounding the NHC, but it is smaller in magnitude than the peak seen in the *R*_1_ RDF. There is also no secondary peak at 9 Å as seen for the reactant, which suggests the absence of longer range structure in the acetate coordination.

**Figure 7:**
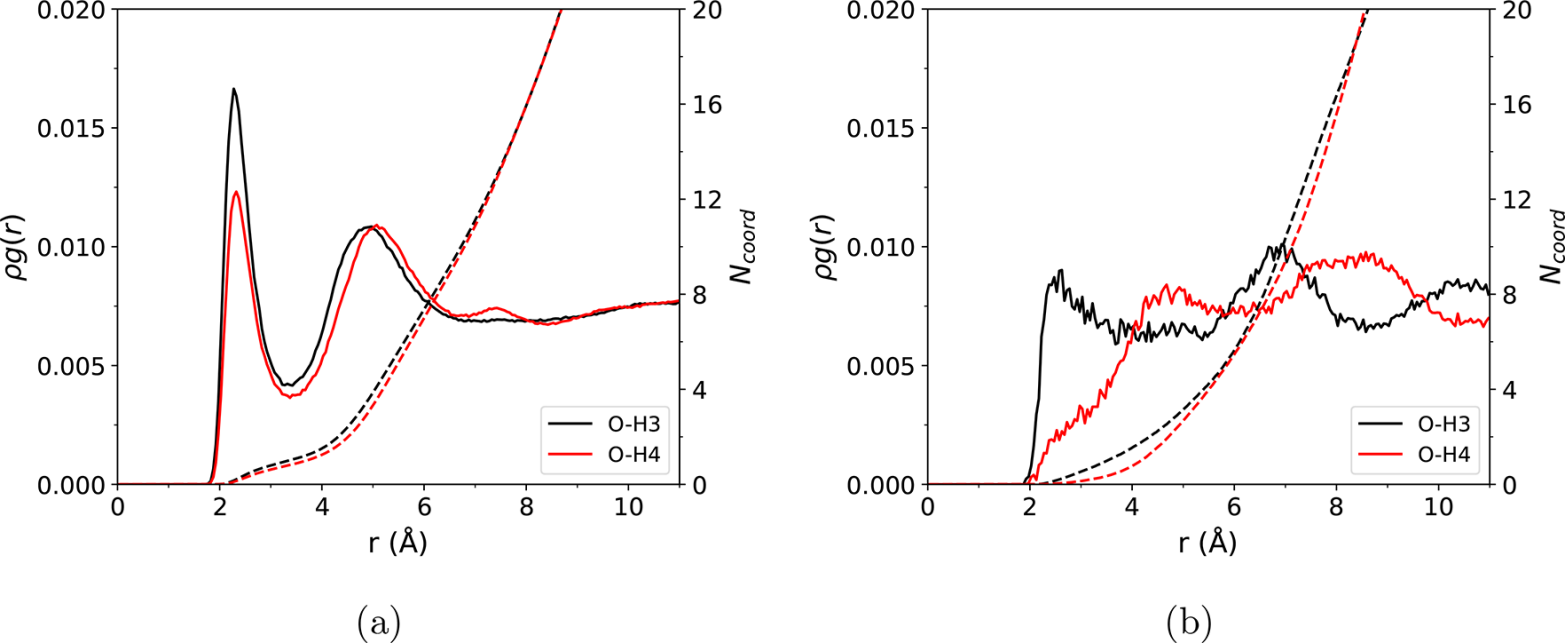
(a) EMIM^+^ H3–O ρ*g*(*r*) and H4–O ρ*g*(*r*). These RDFs were computed using all EMIM^+^ molecules in the simulation. (b) Reacting complex NHC H3–O ρ*g*(*r*) and H4–O ρ*g*(*r*). The corresponding coordination number at a given distance is shown by the dashed lines.

The solvent acetates hydrogen bond to both nonreactive imidazolium protons, but the two hydrogen bonds are unequal in strength. We plot the oxygen–proton RDFs for both nonreactive EMIM^+^ ring protons in Figure 7a, which we label as H3 and H4 (H3 is closer to the methyl group and H4 is closer to the ethyl group; see Figure S20). Figure 7a shows the computed ρ*g*(*r*) with all EMIM^+^ molecules (solvent + reacting complex) in the simulation while Figure S17 plots similar RDFs with only the reacting complex EMIM^+^. The first peak in both RDFs in Figure 7a is due to the oxygen hydrogen bonded directly to the respective proton. There is a stronger hydrogen bond/higher RDF peak at the H3 proton. This is likely due to sterics, with less steric constraint for acetate to hydrogen bond to the proton close to the smaller methyl group than the ethyl group. Interestingly, Figure S17 indicates that the reacting complex EMIM^+^ H4 proton may form a stronger hydrogen bond compared to the H3 proton; however, a definitive conclusion is difficult given the statistical uncertainty.

Upon formation of the NHC, the hydrogen bond strength to these nonreacting imidazolium protons is significantly reduced. Figure 7b shows the NHC nonreactive ring protons–oxygen ρ*g*(*r*); we show the same plot in Figure S19 with the corresponding ρ*g*(*r*) for the reacting complex EMIM^+^. The RDF was computed from a simulation in the “*P*_2_” region of the reaction profile. There is little coordination at the H3 proton, and the H4 proton shows almost no coordination at 2–2.5 Å. This conclusion is consistent with Figure 6, which indicated fewer coordinating acetates around the NHC due to the reduced electrostatics of the neutral molecule. The lower acetate coordination for the NHC leads to less hydrogen bonding with the nonreactive, ring hydrogen atoms.

These RDFs help explain the varying free energies observed in Figure 4a. The solvent stabilizes the ionic *R*_1_ state, as the *P*_2_ state has significantly less coordination around the ring (Figure 6); this inhibits formation of this species through reactions 1 and 2. The RDFs in Figure 7 show that the solvent acetate molecules hydrogen bond to the H3 and H4 EMIM^+^ ring protons, which we discussed earlier in connection with the [EMIM^+^][OAc^–^] bulk liquid and EMIM^+^/(OAc^–^)_2_ trimer free energy profiles in Figure 4. While the OAc^–^ not involved with the initial EMIM^+^ deprotonation is allowed to hydrogen bond to these protons in the gas-phase EMIM^+^/(OAc^–^)_2_ trimer, the solvent prevents this in the liquid phase, leading to the varying free energies and geometries observed in Figures 4 and 5.

### 3.2 Proton-Transfer Free Energy at [EMIM^+^][OAc^–^] Ionic Liquid/Vapor Interface

**Figure 8:**
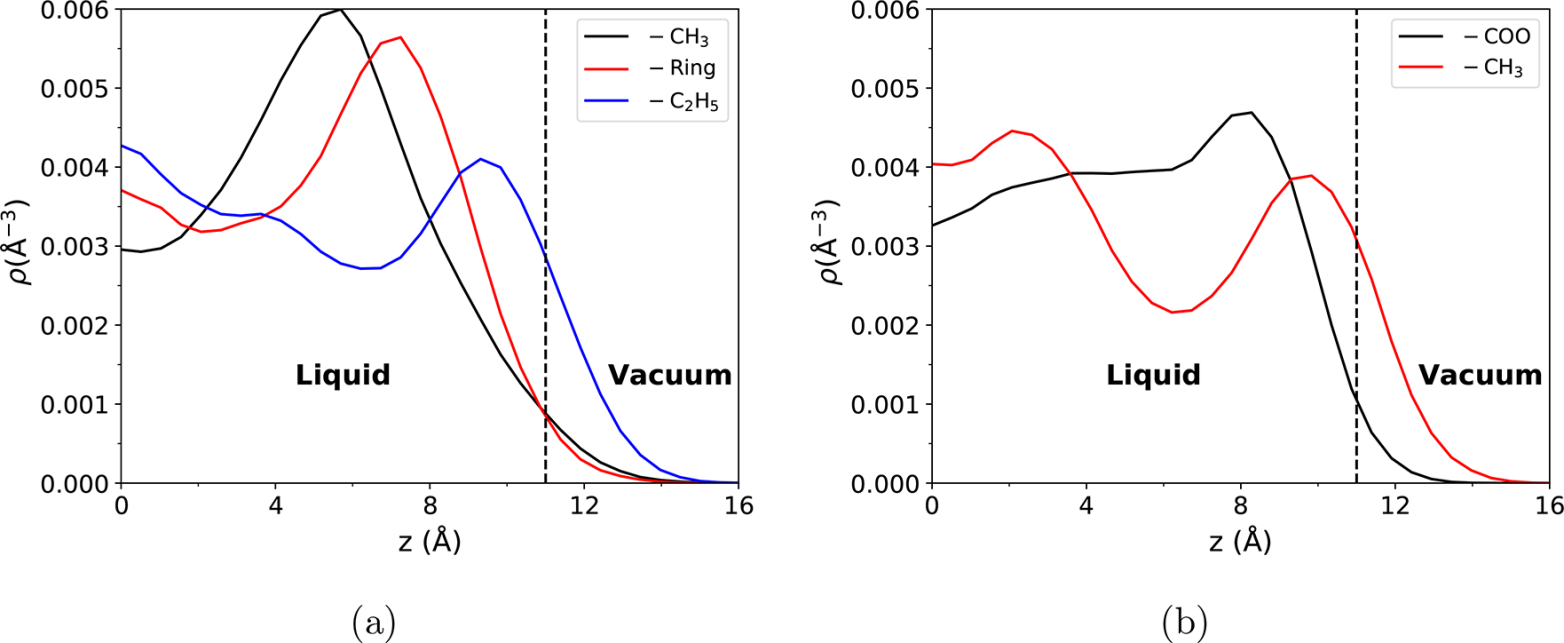
Number density profiles at the [EMIM^+^][OAc^–^] ionic liquid/vapor interface for (a) the center of mass of the EMIM^+^ methyl, ring and ethyl group and (b) the center of mass of the OAc^–^ carboxylate and methyl group. The dashed lines approximately denote the Gibbs dividing surface.

We next analyze the proton-transfer reaction at the [EMIM^+^][OAc^–^] ionic liquid/vapor interface. Because of the important solvation energy contribution to the reaction free energy (as previously discussed), it is interesting to investigate differences between the reaction at the liquid/vapor interface compared to the bulk liquid. Before discussing the interfacial reaction, we first discuss the structure of the ionic liquid/vapor interface. In Figure 8, we plot cation and anion density profiles (decomposed by select functional groups) that span the liquid/vapor interface. As discussed in Section 2.3, we are limited to small system sizes (40 ion pairs), and thus there are significant fluctuations in the interfacial structure; the liquid/vapor density profiles are generated from a 100 ns (nonreactive) simulation for better statistical convergence. We divide the cell into half along the *z* dimension of the simulation box and shift the system center of mass along the *z* dimension to the origin for each frame and average the density profile over both interfaces; this analysis procedure is similar to previous work.^129^Figure 8a shows the number density profiles for the EMIM^+^ methyl group (−CH_3_), ethyl group (−C_2_H_5_), and the imidazolium ring. Figure 8b shows the number density profiles for the OAc^–^ carboxylate (−COO) and methyl (−CH_3_) groups. A dashed line is used to denote the Gibbs dividing surface, which is computed from the total number density profile (Figure S9). Inspection of Figure 8 indicates that nonpolar groups of both the cation and anion have a preference to reside near the vacuum side of the interface. The EMIM^+^ ethyl group is positioned closest to the interface, with the methyl group of EMIM^+^ farther into the liquid side of the liquid/vapor interface. Similarly, the methyl group of acetate is positioned closer to the vacuum while the carboxylate group remains buried in the liquid. Both of these results match with prior simulations of imidazolium ionic liquids, which found the terminal ethyl carbon of various EMIM^+^ cation ILs was oriented toward the vacuum at the liquid/vapor interface;^129,130^ additionally, an AIMD study of [EMIM^+^][OAc^–^] droplets by Brehm and Sebastiani^131^ found that the methyl group of acetate orients itself toward the vacuum.

**Figure 9:**
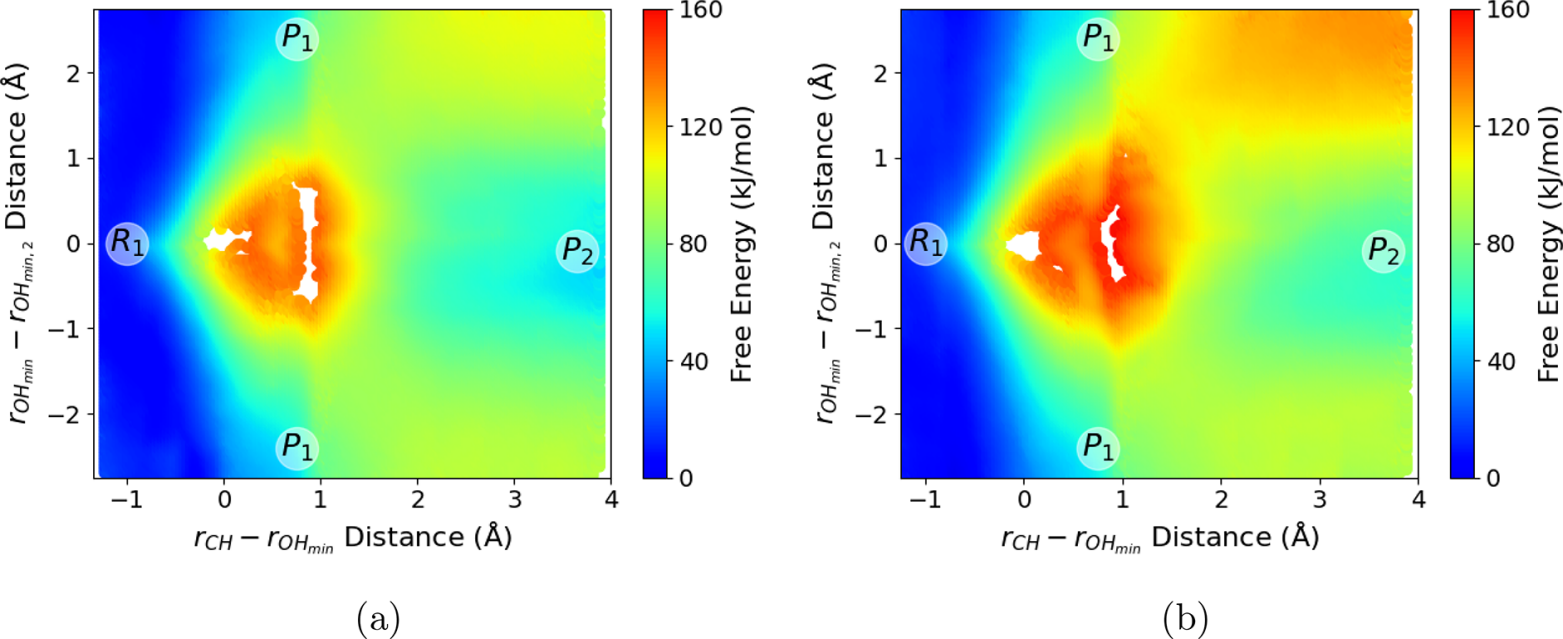
Free energy profiles for the proton-transfer reaction (a) at the [EMIM^+^][OAc^–^] ionic liquid/vapor interface and (b) in bulk [EMIM^+^][OAc^–^] ionic liquid.

We now discuss the free energy of the proton-transfer reactions at the [EMIM^+^][OAc^–^] ionic liquid/vapor interface. As discussed in Section 2.3, this calculation was performed by constraining the reaction complex near the Gibbs dividing surface of the liquid/vapor interface during the free energy calculation, as similar to previously employed procedures.^73^ In Figure 9a, we show the reaction free energy computed at the liquid/vapor interface, as compared to the corresponding reaction free energy previously computed for the bulk ionic liquid (Figure 9b). *A priori*, one might anticipate that the energetics of the reaction at the liquid/vapor interface would be intermediate between that computed for the bulk liquid and the gas-phase ion clusters, based on solvation considerations. However, we (somewhat surprisingly) find that the reaction free energy profile is qualitatively similar as computed for the liquid/vapor interface and the bulk ionic liquid, with only some quantitative differences. The global minimum, which corresponds to the ionic reactants in Figure 1a, is almost identical in the two free energy profiles, and so is the free energy of intermediate “*P*_1_” (Figure 9a). There are, however, quantitative differences between the free energy profiles in the reaction coordinate region spanning “*P*_1_” to “*P*_2_”, which encompasses the transition state and product basins. In general, the proton transfer is better facilitated at the liquid/vapor interface, with a reduced transition state barrier and a lower free energy cost for forming NHC and AcOH/OAc^–^ dimer products; the relative product free energies are ∼60 kJ mol^–1^ at the liquid/vapor interface compared to ∼70 kJ mol^–1^ for the bulk ionic liquid. One reason for the similarity in the reaction free energy profiles is that the reacting complex configurations are much more similar to one another in the liquid and at the liquid/vapor interface compared to the gas-phase EMIM^+^/(OAc^–^)_2_ trimer complex. This is observed in simulation snapshots shown in Figures S11 and S13. In Figure S15, we show histograms of the center of mass NHC/center of mass AcOH/OAc^–^ dimer distance, and it is observed that the histograms largely overlap as computed in the two different environments. As was discussed in Section 3.1, differences in the geometry of the reacting complex lead to higher reaction free energies for the EMIM^+^/(OAc^–^)_2_ trimer complex; these configurational differences are not observed when comparing the reaction at the liquid/vapor and bulk liquid environments. Interestingly, Figure 9 indicates better statistics for the free energy calculation at the liquid/vapor interface, as the reaction profile better matches the required symmetry about CV_2_ = 0 (vertical axis). Evidently, there are different time scales involved with the solvation coordinate(s) within the bulk liquid and liquid/vapor interface, leading to differences in statistical sampling; a more detailed analysis of such solvation time scales is beyond the focus of this work.

**Figure 10:**
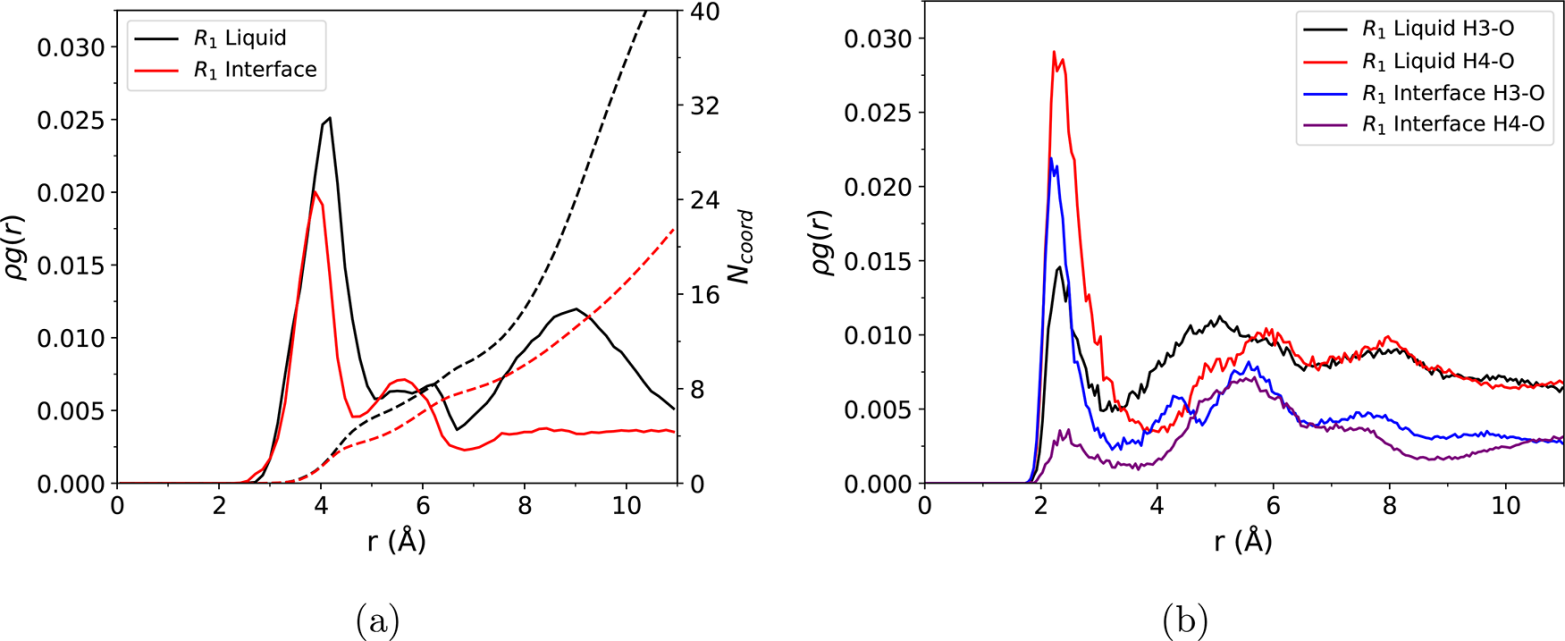
(a) RDFs between reacting complex EMIM^+^ center of mass/acetate oxygen atoms computed for both the liquid and the liquid/vapor interface. (b) RDFs for EMIM^+^ ring nonreactive protons/acetate oxygen atoms computed for both the liquid and the liquid/vapor interface.

**Figure 11:**
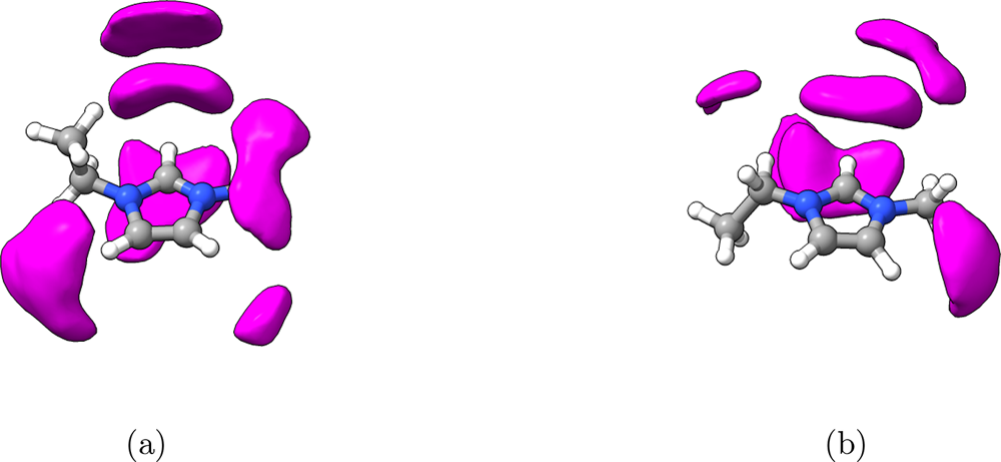
Spatial distribution functions of acetate oxygen atoms surrounding the EMIM^+^ ring for (a) a liquid phase umbrella sampling simulation and (b) a liquid/vapor interface umbrella sampling simulation. Depicted in purple are density regions with isovalue set to 0.532 nm^–3^. These configurations correspond to *R*_1_ regions of the profile.

We analyze the coordination of the reacting complex at the liquid/vapor interface. At short distances, the reacting complex EMIM^+^ at the liquid/vapor interface only has marginally reduced acetate coordination in comparison to the bulk liquid. In Figure 10, we compare the reacting complex EMIM^+^ ring/acetate oxygen RDF at the liquid/vapor interface to that in the bulk liquid. The coordination number is slightly reduced through the first peak in the RDF for the liquid/vapor interface as compared to the bulk ionic liquid. At longer distance, the RDFs and coordination numbers become dissimilar, reflecting the different liquid/vapor and bulk liquid environments. In Figure 10b, we show RDFs between nonreactive EMIM^+^ protons and acetate oxygen atoms, for both the liquid/vapor and bulk liquid environments. In the liquid phase, there are noticeable peaks in the RDF for both of these protons. At the interface, there is a significant peak for the H3 proton (positioned on the side of the imidazolium ring with the methyl group), while there is only a small peak for the H4 proton (positioned on the side of the ring with the ethyl group). As was shown in Figure 8a, the ethyl group of EMIM^+^ is positioned closer to the vacuum than the methyl group, which leads to fewer hydrogen bonds with acetate molecules at the H4 proton. To further demonstrate this distribution of solvent acetates, we show spatial distribution functions (SDFs) in Figure 11 of oxygen atoms surrounding the reactant EMIM^+^ for both environments. These SDFs indicate similar distribution of acetate oxygen atoms around the ring, except at the H4 position which is largely uncoordinated at the liquid/vapor interface.

As discussed, both the transition state and product free energies for the proton-transfer reaction are more favorable/lower by ∼10 kJ mol^–1^ at the liquid/vapor interface compared to the bulk ionic liquid. This is caused by two factors, the first being the reduced solvation around the EMIM^+^ cation at the liquid/vapor interface, which leads to less stabilization and higher free energy of the ionic reactants (*R*_1_ state). The second factor is a direct stabilization of the products at the liquid/vapor interface. Because the product NHC molecule is a neutral species, there is a solvophobic force within the bulk ionic liquid that expels this NHC species to the liquid/vapor interface. This results in an enhanced propensity for NHC at the liquid/vapor interface, corresponding to a more favorable/lower product (“*P*_2_”) free energy in the reaction profile of Figure 9a. To quantify the solvophobic driving force for NHC to reside at the liquid/vapor interface, we compute a potential of mean force (PMF) for the NHC molecule as a function of distance from the liquid/vapor interface. This PMF is shown in Figure S21 and demonstrates that the NHC is stabilized by 15–20 kJ mol^–1^ at the Gibbs dividing surface of the interface relative to its solvation in the bulk ionic liquid. Thus, this solvophobic force is substantial and is an important underlying cause for the quantitative free energy difference of the “*P*_2_” product state in Figures 9a and 9b. To summarize, the proton-transfer reaction is facilitated at the ionic liquid/vapor interface relative to the bulk liquid due to both poorer solvation of the ionic reactants and stabilization of the NHC product molecule at the interfacial environment.

## 4 Conclusion

We have developed a PB/NN reactive force field to simulate proton-transfer reactions of *N*-heterocyclic carbene formation in the [EMIM^+^][OAc^–^] ionic liquid. Reaction free energy profiles were computed for the bulk ionic liquid, its liquid/vapor interface, and additionally EMIM^+^/(OAc^–^)_2_ ion clusters. Our results indicate that the reaction free energy depends on both the geometry/structure of the local reacting complex and the surrounding solvation environment. The EMIM^+^/(OAc^–^)_2_ ion trimer is the smallest cluster for which the proton-transfer reaction proceeds qualitatively (*albeit not quantitatively*) similar to as in the bulk [EMIM^+^][OAc^–^] ionic liquid. This is because the product of the proton-transfer reaction is an NHC species and AcOH/OAc^–^ dimer with a shared proton, and furthermore the third ion electrostatically stabilizes the ionic reactants, reminiscent of the bulk liquid solvation energy; this is distinct from the EMIM^+^/OAc^–^ ion dimer, for which the proton-transfer reaction profile is qualitatively different. There are, however, significant quantitative differences in the reaction free energy profile computed for the EMIM^+^/(OAc^–^)_2_ ion trimer compared to the bulk [EMIM^+^][OAc^–^] ionic liquid. This is due to the constrained reacting complex geometry as well as nonexistent long-range ion solvation for the EMIM^+^/(OAc^–^)_2_ trimer, both of which lead to more favorable NHC formation within the bulk ionic liquid.

In previous literature, there has been debate as the extent of NHC formation/concentration within [EMIM^+^][OAc^–^] and similar ionic liquids. Our computed reaction free energy profiles provide an estimate of the concentration of NHC within the bulk [EMIM^+^][OAc^–^] and at its liquid/vapor interface. Given a reaction free energy of ∼70 kJ mol^–1^ (Figure 4) we estimate that NHC species exist in the bulk [EMIM^+^][OAc^–^] at parts-per-million (ppm) levels; note that such concentrations are catalytically relevant in certain contexts.^3,35^ As mentioned in the Introduction, our PB/NN reactive force field neglects possible proton-sharing/dimerization of the NHC with EMIM^+^ cations. If such interactions are significant, an even higher concentration of NHC would be expected than the ppm levels predicted here. Our results thus support previous conjectures of NHC content in these ionic liquids, which speculated that NHCs are formed in low concentrations but lead to rapid reactions.^53^ We have additionally predicted that NHC concentration is significantly enhanced at the ionic liquid/vapor interface. This is due to both poorer solvation of the ionic reactants as well as solvophobic stabilization of the neutral NHC molecule at the interface. The concentration enhancement at the interface is predicted to be an order of magnitude based on the reaction free energy (Figure 9), with an even larger enhancement predicted based solely on the NHC potential of mean force (Figure S21). The estimated NHC content has important implications for [EMIM^+^][OAc^–^] as a solvent and/or electrolyte, due to the catalytic activity of carbene species. Additionally, the NHC content may affect the chemical/thermal stability of the IL, as the carbene could potentially catalyze decomposition reactions.

We finally comment on the utilization of our PB/NN reactive force field for this study, compared to alternative computational approaches. Ionic liquids are highly viscous with liquid structure consisting of long-range charge oscillations,^132^ such that long simulation times with fairly large simulation boxes are required to minimize statistical uncertainty and finite size effects. While our predictions do exhibit statistical uncertainty (Figure 4) and likely finite size artifacts as well (Section 2.3), our PB/NN reactive force field allows longer simulations for larger systems compared to either AIMD or QM/MM approaches. The key drawback is the significant effort required to parametrize the PB/NN Hamiltonian (Section 2.1). However, similar to related EVB methods, the advantage of PB/NN is that once parametrized, the reactive potential is transferable to arbitrary solvation environments. For example, interesting future directions could include investigation of NHC formation in [EMIM^+^][OAc^–^] mixtures and/or at solid/liquid interfaces. Continued work on improving the accuracy and ease of construction of the PB/NN potentials will be a key route of further research.

## Supporting Information Available

Procedure for generating the AcOH/OAc^–^ training data; *E*_Solute_^Inter,NN^ hyperparameters; *H*_*ij*_ hyperparameters; procedure for generating AcOH *anti* configurations; umbrella sampling details for the AcOH/OAc^–^ dimer; full expressions for diagonal terms in the Hamiltonian and “reinitialization” procedure for the Hamiltonian; PB/NN energy conservation; description of the diabatization; center of mass density profile for the liquid/vapor interface simulations; umbrella sampling procedure for the gas-phase dimer in Figure 2; simulation snapshots from the EMIM^+^/(OAc^–^)_2_/trimer, bulk liquid, and liquid/vapor interface; histograms of the AcOH/OAc^–^ dimer center of mass and the NHC ring center of mass distance; histograms of the angle between the AcOH/OAc^–^ dimer center of mass and the ring plane; spatial distribution functions of the reacting complex EMIM^+^ and oxygen atoms; RDFs of the EMIM^+^ ring protons and oxygen atoms; RDF comparing the EMIM^+^ nonreactive proton/oxygen RDF with the NHC nonreactive proton/oxygen RDF; NHC liquid/vapor interface PMF; order of magnitude estimate for NHC concentration in the bulk liquid and at the liquid/vapor interface (PDF)
Code used to produce the data in this manuscript (ZIP)
