# Supplementary material for: N-Heterocyclic Carbene Formation in the Ionic Liquid [EMIM+][OAc–]: Elucidating Solvation Effects with Reactive Molecular Dynamics Simulations
Source: J Phys Chem B. 2023 Jun 6;127(23):5317–33. doi: 10.1021/acs.jpcb.3c02064 (PMC10278139; doi:10.1021/acs.jpcb.3c02064)
Supplement: Supplementary file 1 — jp3c02064_si_001.pdf [file jp3c02064_si_001.pdf]

Supporting Information: N-Heterocyclic Carbene Formation in the Ionic Liquid [$EMIM^+$][OAc^-]: Elucidating Solvation Effects with Reactive Molecular Dynamics Simulations

John P. Stoppelman and Jesse G. McDaniel*

School of Chemistry and Biochemistry, Georgia Institute of Technology

Atlanta, Georgia, 30332-0400, United States

E-mail: mcdaniel@gatech.edu

Contents

1 Procedure for generating [AcOH][OAc$^-$] dimer training data S4

2 $E_{Solute}^{Inter,NN}$ AP-Net hyperparameters S6

3 H_{ij} AP-Net hyperparameters S8

4 Refinement of $E_{Solute}^{Intra,NN}$ Terms for Acetic Acid *anti* Configuration S10

5 Acetic Acid - Acetate Umbrella Sampling Procedure S11

6 H_{ii} for Multistate Hamiltonian, and "Reinitialization" of Reacting Complex S12

7 PB/NN Energy Conservation S15

8 Diabatization Limitations S19

9 Gibbs Dividing Surface S22

10 Umbrella potentials used for gas phase dimer S23

11 Free energy surface with Labeled Simulation Snapshots S24

12 NHC ring center of mass / AcOH - OAc$^-$ Dimer center of mass distance S28

13 Spatial Distribution Functions S33

14 EMIM$^+$ H3 and H4 ring protons/oxygen RDF S34

15 Ring proton - oxygen RDFs S35

16 Reacting Complex Atom Labeling S37

17 NHC Liquid/Vapor Interface PMF S38

18 Estimate of NHC Concentration in the Bulk Liquid **S39**

19 PB/NN Timings **S41**

References **S43**

1 Procedure for generating [AcOH][OAc$^-$] dimer training data

As mentioned in the main text, we run AIMD simulations with CP2k[S1] to generate training data for proton transfer between acetic acid and acetate. The QUICKSTEP method was used, with the PBE-D3(BJ) exchange-correlation functional and a mixed Gaussian/plane-wave basis set.[S2–S4] The aug-TZVP basis set, a plane-wave cutoff of 280 Rydberg to expand the auxiliary electron density and Goedecker-Teter-Hutter pseudopotentials for the core electrons were used.[S3] SCF convergence criteria was set to 10^{-7} au. NVT simulations were run at temperatures of 300K, 400K, 500K and 600K using a Langevin thermostat with a 0.005 fs^{-1} friction coefficient and a 1 fs timestep. As the proton is shared closely between the two acetate molecules during the gas-phase AIMD simulations, three additional simulations were run at 300K with harmonic umbrella potentials. This allows for sampling dimer geometries at greater intermolecular separations. The umbrella potentials were placed between an oxygen atom on one molecule and the reactive proton. Each umbrella potential ($\frac{1}{2}kx^2$) used a 200.0 kJ/mol/Å^2 force constant and were centered at values of 3, 3.25 and 3.5 Å.

The AIMD simulations yielded approximately 290,000 acetic acid/acetate dimers. We computed SAPT0/aug-cc-pVTZ energies for individual configurations with Psi4.[S5] As this is a symmetric reaction, two SAPT0 calculations were performed for each geometry: one with the acetic acid classified as the molecule with the shorter minimum O–H bond distance and one with the acetic acid classified as the molecule with the longer minimum O–H bond distance. This yields 580,000 SAPT0 calculations. These were used to train the $E^{Inter,NN}_{Solute}$ neural network. We use AP-Net as in our prior work for the neural network architecture.[S6,S7] Details of the AP-Net neural network hyperparameters and training details are described in the next section. As the $E^{Inter,NN}_{Solute}$ term is a residual correction to $E^{Nonbonded,FF}_{Solute,Solvent}$, we plot $E^{Inter,NN}_{Solute} + E^{Nonbonded,FF}_{Solute,Solvent}$ vs. SAPT0 - δ_{HF} energies for a subset of the test set in Figure S1. The mean absolute error (MAE) for the total test set is 0.55 kJ mol^{-1}. As mentioned

previously, $E_{Solute}^{Inter,NN}$ is multiplied by a damping function. We use the Fermi-Dirac function for this term; the parameters are shown in Table S1.

2 $E_{Solute}^{Inter,NN}$ AP-Net hyperparameters

The AP-Net architecture is used for modeling the $E_{Solute}^{Inter,NN}$ term. As in our prior work,[S6] the interaction energy predicted by AP-Net is modified slightly from the published definition by Glick et al.[S7] The pairwise energy and sum of pairwise energies are multiplied by separate damping functions, as shown in Equations S1-S3:

$$E_{Solute}^{Inter,NN} = \sum_{i \in A, j \in B} (E_{ij} f_c(r_{ij})) f(r_{bond}) \tag{S1}$$

$$f_c(r_{ij}) = \begin{cases} \frac{1}{2}(cos(\frac{\pi r_{ij}}{r_c}) + 1) & r_{ij} \leq r_c \\ 0 & r_{ij} > r_c \end{cases} \tag{S2}$$

$$f(r_{bond}) = \frac{1}{(e^{\beta(r_{bond} - \mu r_o)} + 1)} \tag{S3}$$

The AP-Net sum runs over atoms on monomers A and B. In Equation S1, the $f_c(r_{ij})$ is a standard cutoff function used in many neural network frameworks.[S8] The r_c cutoff is set to 4.0 Å, which ensures that there is no contribution to the total interaction energy by atom pairs separated by more than this amount. This restricts $E_{Solute}^{Inter,NN}$ to serve as a short-range correction. The other cutoff function, $f(r_{bond})$, is a Fermi-Dirac function. The function takes as input the length of the dissociating bond. If the bond stretches too far, then the contribution to the total energy from the diabat is small. The Fermi-Dirac function zeros out the energy of the neural network at these distances. The parameters for the cutoff function are shown in Table S1. The r_o term is listed as the equilibrium bond length for the bond in question, and μ is an arbitrary factor that scales it by some amount. The procedure for selecting μ involves determining where the difference between the diabat and reference energy becomes zero, and is explained further in the Supporting Information of Stoppelman and McDaniel[S6]

Table S1: Fermi-Dirac function parameters used for $E^{Inter,NN}_{Solute}$.

Neural Network	β (Å^{-1})	μ	r_o (Å)
$E^{Inter,NN}_{Solute}$	30.0	1.86	0.97

The hyperparameters we use for training $E^{Inter,NN}_{Solute}$ for the acetic acid/acetate dimer are similar to our prior work and to Glick et al.[S7], and we refer the reader to there for information about the form of the atomic centered symmetry functions (ACSFs) and atomic pair symmetry functions (APSF). We use 43 ACSFs and 21 APSFs. The shifted softplus network from SchNet was used as the activation function.[S9] The loss function is the mean squared error (MSE) loss function shown in Equation S4. The loss is minimized between the (SAPT0 - δ_{HF}) - SAPT-FF energies that make up the training data and the neural network prediction.

$$\mathcal{L} = \|E - \hat{E}\|^2 \tag{S4}$$

For training the neural network, we use a train-validation-test split of 80:15:5. The neural network is trained using the Adam optimizer, with an initial learning rate of 5.0 x 10^{-4} and a decay rate of 0.8.[S10] Training finished once the learning rate decayed to a value of 1.0 x 10^{-6}. We use batch sizes of 100 for both training and validation. The mean absolute error (MAE) for $E^{Inter,NN}_{Solute}$ on the test set is shown in Figure S1.

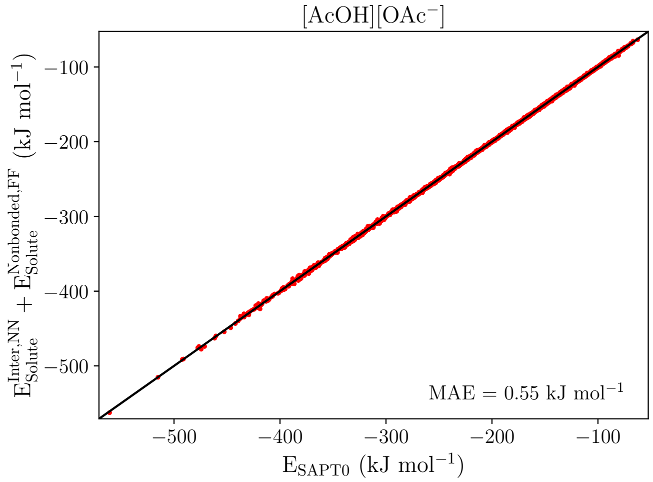

Figure S1: $E_{Solute}^{Inter,NN} + E_{Solute,Solvent}^{Nonbonded,FF}$ vs. SAPT0/aug-cc-pVTZ for a subset of the test set (approximately 3000 geometries).

3 H_{ij} AP-Net hyperparameters

The modified version of AP-Net still models pairwise energies between atoms, but the restriction of the pairs being on opposite monomers is removed.

$$H_{12} = (|\sum_{i,j\neq i} E_{i,j}|) f_c(r_{\min OH,1}) f_c(r_{\min OH,2}) \tag{S5}$$

The Fermi-Dirac functions take as input the minimum O–H bond distance on each acetate/acetic acid molecule. The parameters for the Fermi-Dirac functions are shown in Table S2. We use structures from the assembled acetic acid/acetate training set that have bond lengths less than 2.0 Å(which is equal to $\mu * r_o$ in Equation S3, taking μ and r_o as the values from Table S2); the damping functions prevent the H_{ij} term from making predictions for structures with O–H bonds longer than 2.0 Å. The hyperparameters are the same as for training $E_{Solute}^{Inter,NN}$ for acetic acid/acetate. As we include forces this time, the loss function is modified:

$$\mathcal{L} = \rho \|E - \hat{E}\|^2 + (1-\rho)\frac{1}{3N}\sum_{i=0}^{N} \|\mathbf{F_i} - \hat{\mathbf{F}}_\mathbf{i}\|^2 \tag{S6}$$

ρ is set to 0.1 here.

Table S2: Fermi-Dirac function parameters used for H_{ij} for acetic acid-acetate proton transfer.

Neural Network	β (Å^{-1})	μ	r_o (Å)
H_{ij}	30.0	2.06	0.97

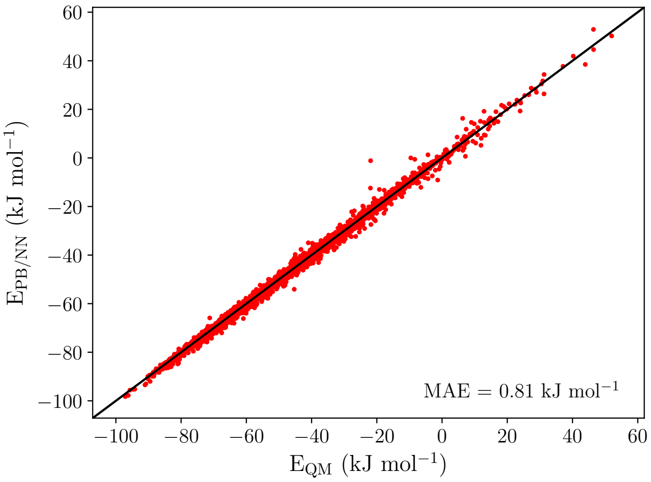

Figure S2: PBE-D3(BJ)/aug-cc-pVTZ energies vs the force field $H_{PB/NN}$ energies with both O–H bond distances less than 2.0 Å. The plot shows 5000 randomly-selected structures from the test set; the MAE listed corresponds to the full test set.

4 Refinement of $E_{Solute}^{Intra,NN}$ Terms for Acetic Acid *anti* Configuration

For condensed phase simulations, we found it necessary to modify the $E_{Solute}^{Intra,NN}$ acetic acid term from its original parameterization.[S6] In the gas-phase, the *syn* configuration of acetic acid is the stable form; however, in water, the *syn* configuration has been found to be approximately isoenergetic with the *anti* configuration.[S11] We find that this is true for the bulk ionic liquid as well. As our neural networks were built with structures mainly taken from gas-phase AIMD simulations, the *anti* configuration was not properly represented in each neural network's training set. In order to add these structures to the various training sets, we ran gas-phase AIMD simulations of the NHC/acetic acid dimer and the acetic acid-acetate dimer with the acetic acid dihedral restrained to various values from 0° to 180°. We took approximately 50,000 acetic acid configurations from these simulations, computed PBE-D3(BJ)/aug-cc-pVTZ energies/forces and added them to the $E_{Solute}^{Intra,NN}$ acetic acid training set; training details for this can be found in the prior work.[S6] SAPT0/aug-cc-pVTZ calculations were then run for both NHC/acetic acid and acetic acid/acetate dimers (again approximately 50,000 structures for both) and then both $E_{Solute}^{Inter,NN}$ neural networks were retrained. Finally, H_{12} and H_{23} were both retrained with DFT energies and forces on the collected dimers. We find that this is sufficient for modeling the varying acetic acid dihedral angle configurations in the reaction.

5 Acetic Acid - Acetate Umbrella Sampling Procedure

We employ umbrella sampling to produce a 2D free energy surface for proton transfer between acetic acid and acetate. The collective variable we used is shown in Equation S7:

$$s = \min r_{O_1H}, r_{O_2H} \tag{S7}$$

This is the minimum distance between the oxygens on one molecule with the reactive proton. This CV is computed for both molecules in order to construct the free energy surface. Umbrella sampling simulations were run for both AIMD and PB/NN using the Hamiltonian described in Section 2.1 of the main text. The AIMD simulations were run using the CP2k/ASE interface,[S1,S12] and the PB/NN simulations were also run with ASE. Plumed was used to apply the harmonic umbrella potentials.[S13] A 200.0 kJ/mol/Å^2 force constant was used for each umbrella ($\frac{1}{2}kx^2$). The umbrella potentials were centered at values shown in Table S3. Each window was run for approximately 20 ps. The free energy surfaces are shown in Figure 2 in the Main Text.

Table S3: List of windows used for umbrella sampling for the acetic acid-acetate dimer in the gas phase. The distances are given in Å.

min O-H Distance molecule 1	min O-H Distance molecule 2
1.0	2.0
1.1	1.9
1.2	1.8
1.3	1.7
1.4	1.6
1.5	1.5
1.6	1.4
1.7	1.3
1.8	1.2
1.9	1.1
2.0	1.0

6 H_{ii} for Multistate Hamiltonian, and "Reinitialization" of Reacting Complex

We explicitly list each term in the diagonal components of the Hamiltonian, making clear which molecules in the reacting complex the neural networks are applied to.

$$H_{11} = E^{Morse}_{EMIM^+} + E^{Intra,NN}_{EMIM^+} + E^{Intra,NN}_{OAc^-,1} + E^{Intra,NN}_{OAc^-,2} + \\ E^{Bonded,FF}_{Solvent} + E^{Inter,NN}_{EMIM^+/OAc^-,1} + E^{Inter,NN}_{EMIM^+/OAc^-,2} + E^{electronic}_{Solute} \tag{S8}$$

$$H_{22} = E^{Morse}_{AcOH} + E^{Intra,NN}_{NHC} + E^{Intra,NN}_{AcOH} + E^{Intra,NN}_{OAc^-,2} + \\ E^{Bonded,FF}_{Solvent} + E^{Inter,NN}_{NHC/AcOH} + E^{Inter,NN}_{AcOH/OAc^-,2} + E^{electronic}_{Solute} \tag{S9}$$

$$H_{33} = E^{Morse}_{AcOH} + E^{Intra,NN}_{NHC} + E^{Intra,NN}_{OAc^-,1} + E^{Intra,NN}_{AcOH} + \\ E^{Bonded,FF}_{Solvent} + E^{Inter,NN}_{NHC/AcOH} + E^{Inter,NN}_{AcOH/OAc^-,1} + E^{electronic}_{Solute} \tag{S10}$$

In the main text, we discussed the issue of "reinitializing" the PB/NN Hamiltonian when the reactive complex identity changes during the course of the simulation. This occurs when a solvent acetate ion "OAc_s^-" displaces a closer contact acetate ion "OAc_2^-" in the reacting complex. Here, we provide more detail for how the PB/NN Hamiltonian is reinitialized in this case.

First, the E^{Morse}_{Solute} term in H_{33} applied to the OAc_2^- O–H bond must be reapplied to the OAc_s^- O–H bond. OAc_s^- approaching closer to the proton than OAc_2^- implies a large O–H bond distance. The large bond distance makes H_{33} a high-energy state in comparison to H_{11}

and H_{22}, and the coupling elements with H_{33} to the other states will be close to zero. Thus, while redefining E_{Solute}^{Morse} from OAc_2^- to OAc_s^- does have an artificial effect on this specific term, it has no effect on the ground-state energy of the system.

Next, the $E_{Solute}^{Intra,NN}$ neural networks within each diabat need to be redefined. In H_{11} and H_{22}, the acetate $E_{Solute}^{Intra,NN}$ model is applied to OAc_2^-, and in H_{33} the acetic acid $E_{Solute}^{Intra,NN}$ model is applied to OAc_2^-. As H_{11} and H_{22} are both contributing to the ground-state energy, we find that removing the $E_{Solute}^{Intra,NN}$ model on OAc_2^- and applying it to OAc_s^- has a significant effect on the ground-state energy. This is because the intramolecular components of OAc_s^- are described by $E_{Solvent}^{Bonded,FF}$, and in this process we would have to turn off this term and turn on the acetate $E_{Solute}^{Intra,NN}$ term for this molecule. There is essentially no way to parameterize $E_{Solute}^{Bonded,FF}$ so that the intramolecular configurations predicted by this term are close to those predicted by $E_{Solute}^{Intra,NN}$, leading to a jump in energy by switching the two terms. In order to avoid this, we initialize the simulation so that each diabatic state has the acetate $E_{Solute}^{Intra,NN}$ term applied to the 4 closest solvent acetates to the reacting proton. This means that the $E_{Solute}^{Intra,NN}$ term does not need to be reinitialized in H_{11} and H_{22}, as both OAc_2^- and OAc_s^- are modeled using it. In H_{33}, the acetic acid $E_{Solute}^{Intra,NN}$ term on OAc_2^- needs to be reapplied to OAc_s^- and the acetate $E_{Solute}^{Intra,NN}$ on OAc_s^- needs to be applied to OAc_2^-; again, since H_{33} is a high-energy state, this has no consequence on the ground-state energy.

The various $E_{Solute}^{Inter,NN}$ terms within each diagonal element of the Hamiltonian also need to be switched in this case. In H_{11}, the $E_{Solute}^{Inter,NN}$ $EMIM^+$ /acetate model between OAc_2^- and $EMIM^+$ needs to be reapplied to OAc_s^- and $EMIM^+$; in H_{22}, the $E_{Solute}^{Inter,NN}$ AcOH/acetate model between OAc_2^- and OAc_1^- needs to be reapplied to OAc_s^- and OAc_1^-; in H_{33}, the $E_{Solute}^{Inter,NN}$ NHC/AcOH model between OAc_2^- and the NHC needs to be reapplied to OAc_s^- and the NHC. Additionally, in H_{33}, the $E_{Solute}^{Inter,NN}$ AcOH/acetate model again needs to be reapplied from OAc_2^- and OAc_1^- to OAc_s^- and OAc_1^-. Switching these terms within H_{11} and H_{22} unfortunately does have an effect on the ground-state energy. In order to avoid this, we would have to damp the $E_{Solute}^{Inter,NN}$ terms so that they only predict nonzero values at close

distances. Currently, the $E_{Solute}^{Inter,NN}$ pairwise energies are multiplied by a cutoff function that takes effect at separations of 4 Å (see Equation S1); this would need to be shortened to 2 Å or less in order to avoid having the “switching” procedure affect the total ground-state energy. We find that this would hinder the ability of the $E_{Solute}^{Inter,NN}$ models to function as a correction to the $E_{Solute,Solvent}^{Nonbonded,FF}$, so we do not adjust the cutoff. The fluctuations in the total energy caused by the switching procedure are on the order of 10 kJ mol^{-1} and can be observed in Figures S4d and S5d.

The last set of terms that would need to be adjusted is the coupling terms. In this scenario, the H_{13} and H_{23} coupling terms would need to be switched from OAc_2^- to OAc_s^-. Again, since the separation between OAc_2^- and the reacting proton is large, the value of both coupling elements will be close to zero and thus switching them between molecules will have no effect on the total energy.

7 PB/NN Energy Conservation

Here we show the energy conservation of the PB/NN Hamiltonian, comparing it to a standard simulation. Figure S3 shows the extent of energy conservation for a nonreactive NVE simulation of 40 [EMIM$^+$][OAc$^-$] pairs. Standard force field terms are used for the intramolecular components, and the SAPT-FF force field is used for the intermolecular terms. The simulation is run using ASE calling OpenMM as a calculator. Due to the Drude SCF procedure, there is an energy drift of approximately 16 kJ mol^{-1} ps^{-1}. In Figures S4 and S5, we show the PB/NN energy conservation for different points along the reaction profile shown in Figure 2 of the main text. Figure S4a and Figure S4b show the energy conservation for windows with umbrella potential values of $\Delta(CH - OH_{min}) = -1.0$ Å and $\Delta(OH_{min,1} - OH_{min,2}) = 0.0$ Å. Figure S4c and Figure S4d show the energy conservation of windows with umbrella potential values of $\Delta(CH - OH_{min}) = -1.0$ Å and $\Delta(OH_{min,1} - OH_{min,2}) = 2.5$ Å. Note that Figure S4a-b essentially show the same extent of energy conservation as Figure S3. This is because the proton is localized on EMIM$^+$, and thus the ground-state energy is equal to H_{11}. Also, the umbrella potential fixes the acetates to be close to equidistant to the reactive proton, meaning that the switching procedure does not need to be employed for this window. Figure S4c-d show various jumps in the total energy due to the switching procedure for $E^{Inter,NN}_{EMIM^+/OAc^-}$ as acetates from the solvent may come closer than the acetate in the reacting complex. Figure S5a-b show the energy conservation for a simulation with umbrella potential values of $\Delta(CH - OH_{min}) = 3.5$ Å and $\Delta(OH_{min,1} - OH_{min,2}) = 0.0$ Å. Note for these windows the ground-state energy will be a mix of H_{22} and H_{33}, as the proton is shared equally between an acetic acid molecule and acetate. This leads to slightly worse energy conservation than seen in Figure S3 and Figure S4a-b. Figure S5c-d show the energy conservation for a simulation with umbrella potential values of $\Delta(CH - OH_{min}) = 3.5$ Å and $\Delta(OH_{min,1} - OH_{min,2}) = 2.5$ Å. The switching procedure is applied again here, as it is possible for solvent acetates to approach closer than the acetate considered part of the reacting complex. The jumps in energy are smaller in this case, as now the $E^{Inter,NN}_{AcOH/OAc^-}$

model is switched between acetate molecules. These are essentially limiting cases of the energy conservation performance; Figure S4a-b represent the best possible performance for the Hamiltonian for windows where the acetates are placed equidistant to the proton. Figure S4c-d places the secondary acetate as far away from the proton as is possible for our reaction profile, so this would lead to the largest number of events that requires switching acetates in and out of the Hamiltonian. Every other window would exhibit energy conservation intermediate to these two simulations. All these fluctuations are smaller than those caused by the NVT thermostat, but we do acknowledge that the procedure may cause some (relatively minor) artifacts.

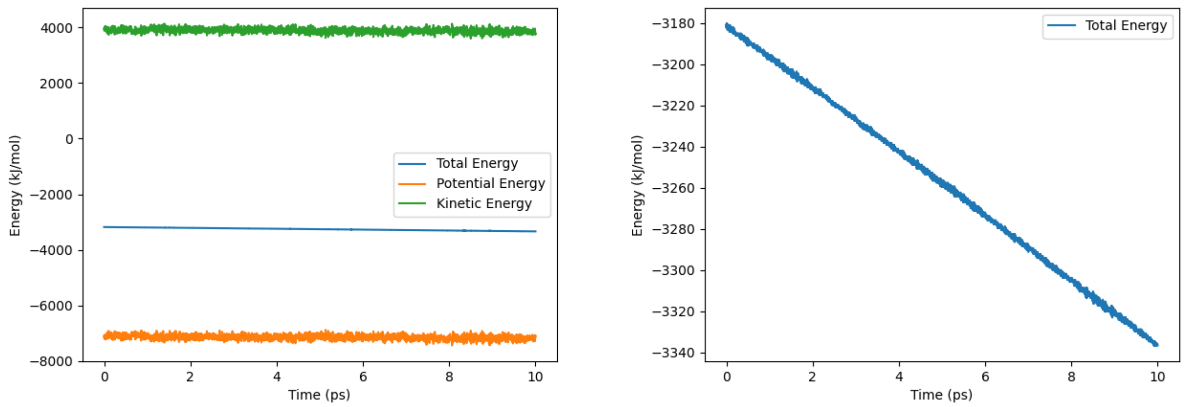

(a) (b)

Figure S3: Energy conservation from a standard simulation of 40 [EMIM$^+$][OAc$^-$]pairs. a) Shows the total, kinetic and potential energy while b) shows only the total energy on a closer scale.

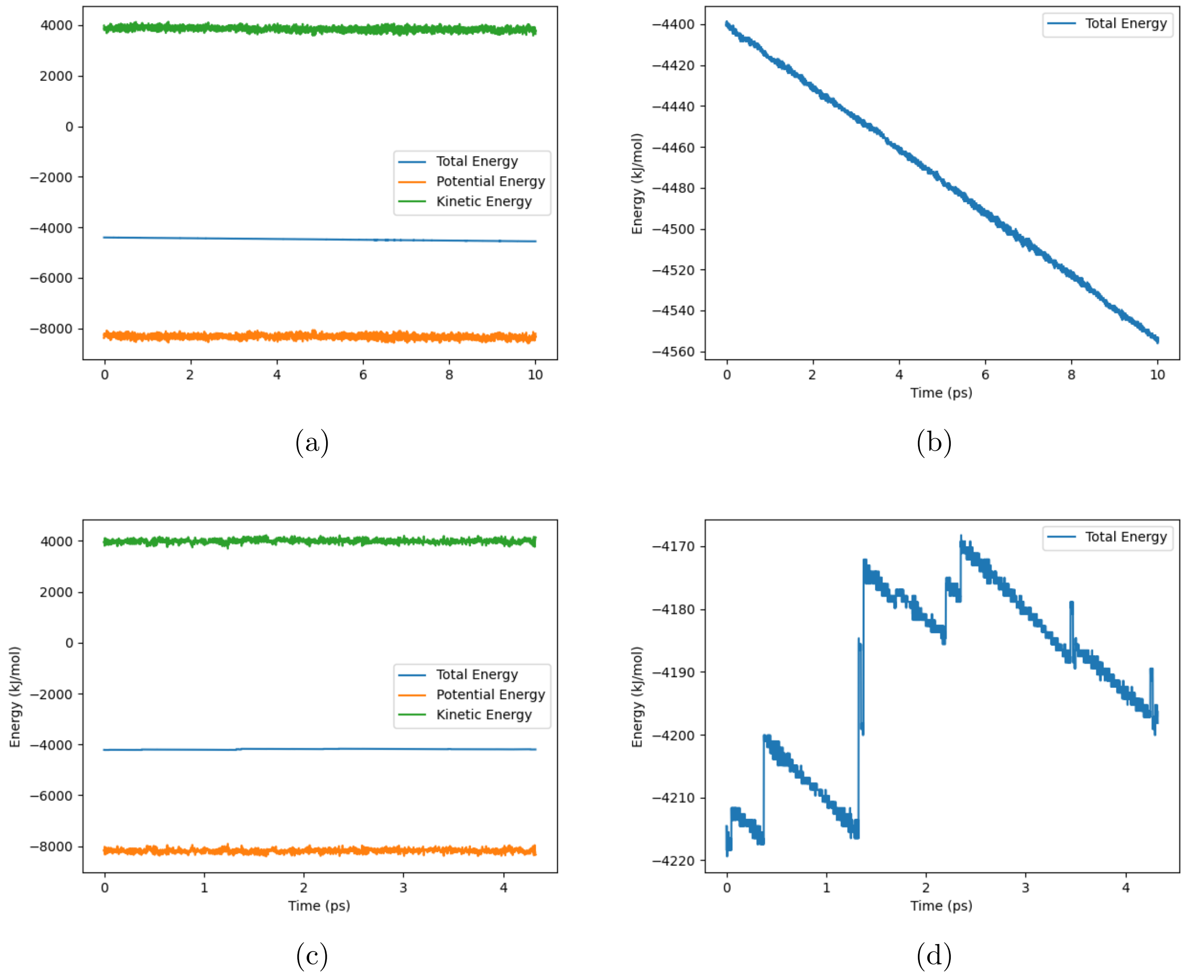

Figure S4: Energy conservation for umbrella sampling potentials of $\Delta(CH - OH_{min})$ = -1.0 Å. The secondary umbrella potential, $\Delta(OH_{min,1} - OH_{min,2})$, is set to 0.0 Å for a) and b). For c) and d), this umbrella potential is set to 2.5 Å.

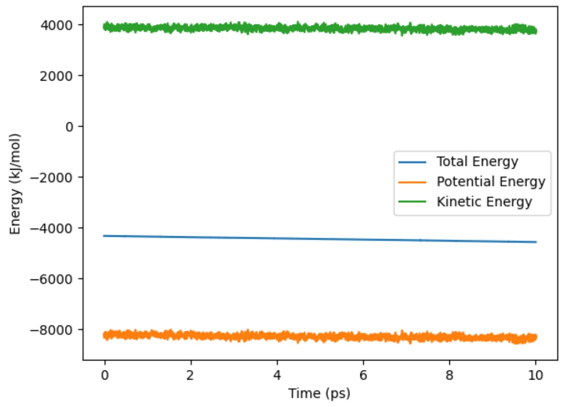

(a)

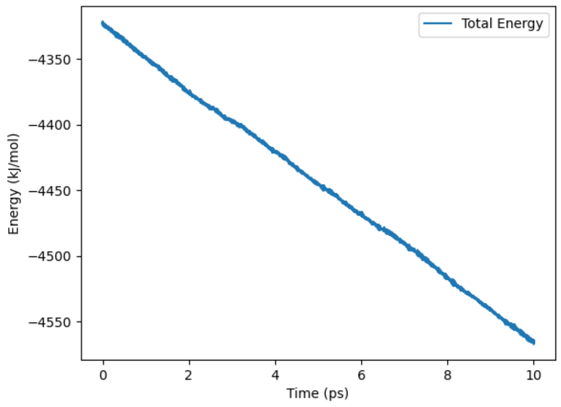

(b)

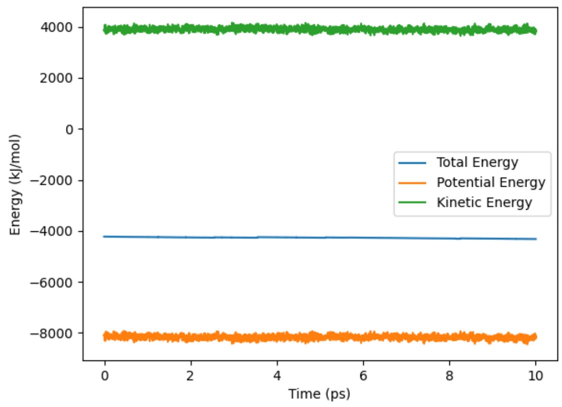

(c)

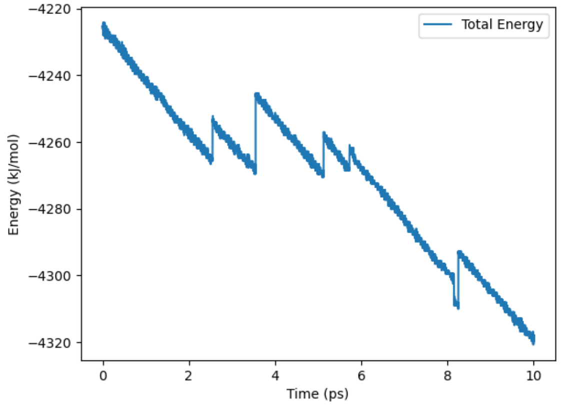

(d)

Figure S5: Energy conservation for umbrella sampling potentials of $\Delta(CH - OH_{min}) = 3.5$ Å. The secondary umbrella potential, $\Delta(OH_{min,1} - OH_{min,2})$, is set to 0.0 Å for a) and b). For c) and d), this umbrella potential is set to 2.5 Å.

8 Diabatization Limitations

As we noted in our previous work, the current scheme for the diabats means that the intermolecular interactions for the reacting complex change from a DFT description to SAPT0 - δ_{HF} description as the reaction occurs. The region where the switch between the two levels of theory happens is determined by the Fermi-Dirac functions applied to the H_{ij} terms. There is some ambiguity in determining the center for the Fermi-Dirac function, although we aim for the point along the reaction coordinate for which the difference between DFT and SAPT0 - δ_{HF} interaction energies is close to zero. Unfortunately, there is no guarantee that DFT and SAPT0 - δ_{HF} will agree for every set of configurations or that they will agree after the difference becomes zero. We show potential energy scans in Figure S6-S8 from DFT (PBE-D3(BJ)/aug-cc-pVTZ), DFT monomer energies (PBE-D3(BJ)/aug-cc-pVTZ) + SAPT0/aug-cc-pVTZ interaction energies, DFT monomer energies (PBE-D3(BJ)/aug-cc-pVTZ) + SAPT0 - δ_{HF}/aug-cc-pVTZ (our diabat definition) and PB/NN; the scans were constructed from optimized geometries at the PBE-D3(BJ)/aug-cc-pVTZ level of theory. The geometry optimizations were performed using the geomeTRIC software package interfaced with Psi4.[S5,S14] In Figure S6 a EMIM$^+$ /acetate dimer is modeled; in Figure S7 a NHC/acetic acid dimer is modeled; and in Figure S8 an acetic acid/acetate dimer is modeled. Asymptotically, the energies eventually agree with one another, but there is a significant discrepancy at intermediate distances. One could increase the range where H_{ij} is applied until DFT and SAPT0 - δ_{HF} interaction energies agree asymptotically, but this would delay switching to the pure diabatic state. Physically reasonable diabat definitions are important for modeling solvent effects on chemical reactions in these methods. The differing levels of theory do cause a sudden increase in the potential energy at the transition to the pure diabat, which can be seen in Figures 3, 4 and 9 in the Results section and also Figures S6-S8; although the transition is smooth due to the damping functions, this represents an unphysical artifact on the simulations. As we are focused on environmental effects on reactions 1 and 2 in this work, this artifact fortunately does not affect this analysis, as it occurs regardless

of environment; however, it does impact the quantitative results. We treat our results as semi-quantitative, and leave determining a more rigorous/effective diabatization procedure for further work. We do slightly extend the Fermi-Dirac damping function for $E^{Inter,NN}_{Solute}$ between EMIM$^+$ /acetate and the Fermi-Dirac damping function for H_{12} that takes the EMIM$^+$ C–H bond distance as input (see Equation S5 for the form of the coupling terms). This extends the point along the reaction for which the Hamiltonian transitions to the pure H_{22} state, which slightly decreases the change in potential energy at the transition, as shown in Figure S8. The new vs. old damping parameters are shown in Table S4.

Table S4: Old and new parameters for the Fermi-Dirac function used for $E^{Inter,NN}_{EMIM^+/OAc^-}$.

Molecule	β (Å^{-1})	μ	r_o (Å)
$E^{Inter,NN}_{EMIM^+/OAc^-}$ Old	30.0	1.75	1.0847
$E^{Inter,NN}_{EMIM^+/OAc^-}$ New	30.0	1.84	1.0847

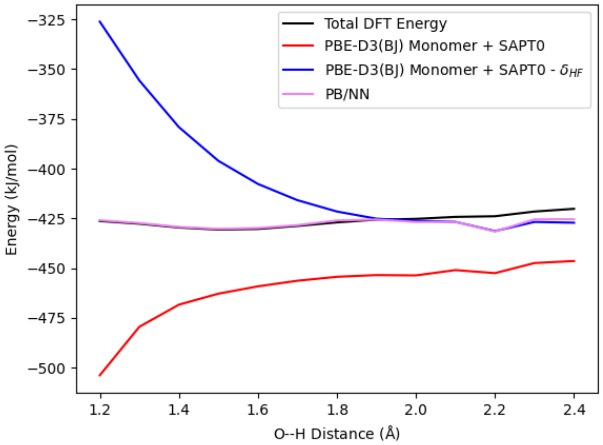

Figure S6: Comparison of the total DFT energy, monomer DFT energy + SAPT0 energy, monomer DFT energy + SAPT0 - δ_{HF} energy and PB/NN energy for [EMIM$^+$][OAc$^-$]. The energies are plotted with respect to the distance from the closest acetate oxygen to the reactive proton.

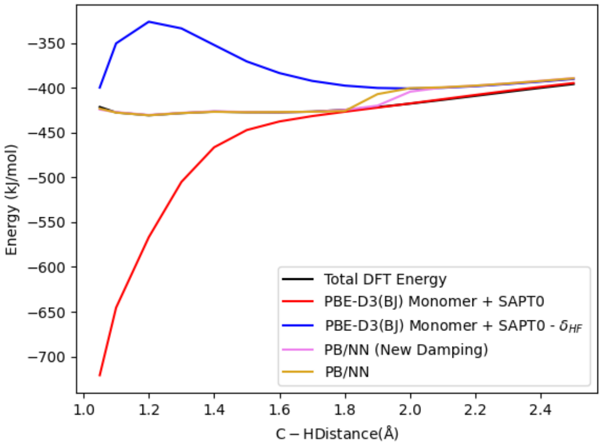

Figure S7: Comparison of the total DFT energy, monomer DFT energy + SAPT0 energy, monomer DFT energy + SAPT0 - δ_{HF} energy and PB/NN energy for NHC/AcOH. Note the difference between the relaxed, isolated gas-phase monomer energies of NHC/Acetic acid and EMIM$^+$ /acetate is added to the PBE-D3(BJ) monomer + SAPT0 and PBE-D3(BJ) monomer + SAPT0 - δ_{HF} terms. The energies are plotted with respect to NHC carbon-proton distance.

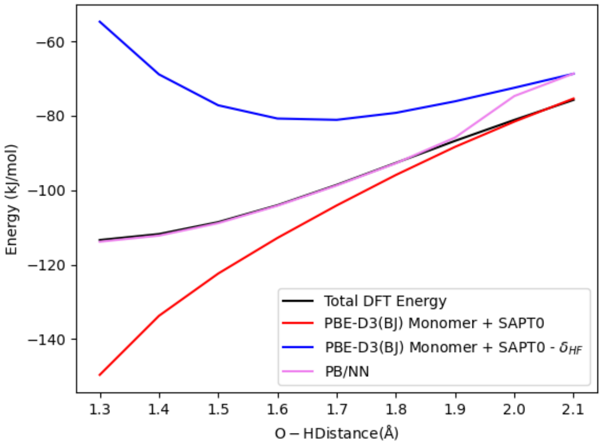

Figure S8: Comparison of the total DFT energy, monomer DFT energy + SAPT0 energy, monomer DFT energy + SAPT0 - δ_{HF} energy and PB/NN energy for AcOH/OAc$^-$. The energies are plotted with respect to the distance from the closest acetate oxygen to the reactive proton.

9 Gibbs Dividing Surface

We plot the number density profile along the z-dimension for a 100 ns liquid/vapor interface (nonreactive) simulation, using the OpenMM settings described in the main text. We count the number of ions (separately for cations and anions) within discretized bins along the z-axis; we use the center of mass for each ion belonging to each type for the binning procedure. We divide the simulation cell in two and average the results from the center of the cell. Note that the liquid is placed at the center of the unitcell and there is a vacuum gap on both sides. The dimensions of the cell are 22.08 Å x 22.08 Åx 66.24 Å. The Gibbs dividing surface occurs where the density falls to approximately half that of the bulk value, which is approximately 11 Å from the center of the simulation cell in this case.

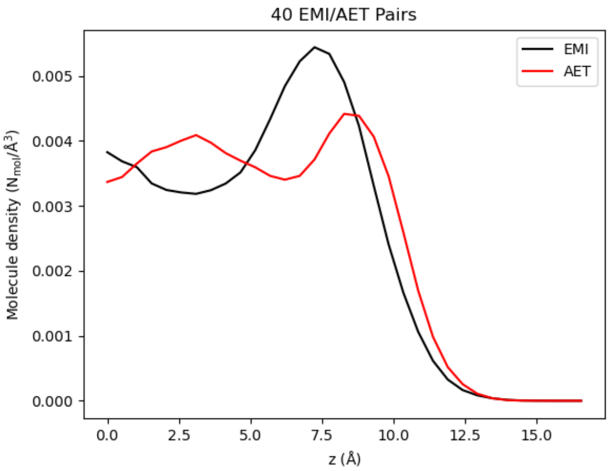

Figure S9: Number density profile for both $EMIM^+$ and acetate at the air-liquid interface.

10 Umbrella potentials used for gas phase dimer

Umbrella sampling for the gas phase dimer was performed using r_{CH} and $r_{OH_{min}}$ (the minimum oxygen-proton distance) as the collective variables; the center umbrella sampling windows are listed in Table S5 and a force constant of 200.0 kJ/mol/Å^2 was used for all umbrella potentials ($\frac{1}{2}kx^2$). These simulations were run with a 2x2 Hamiltonian (upper 2x2 block of Equation 3 in the Main Text), as there is no third reacting species for the dimer.

Table S5: List of windows used for umbrella sampling of the gas phase dimer. The values of the C-H and O-H distances (in Å) that the umbrella potentials were centered at are listed.

C-H Distance	O-H distance
1.00	2.50
1.05	2.40
1.10	2.30
1.15	2.20
1.15	2.10
1.20	2.00
1.20	1.90
1.25	1.80
1.25	1.70
1.25	1.60
1.25	1.50
1.25	1.40
1.25	1.30
1.30	1.30
1.40	1.25
1.50	1.25
1.60	1.25
1.70	1.10
1.80	1.10
1.90	1.05
2.00	1.05
1.95	1.00
1.95	1.05
1.95	1.10
2.00	1.00
2.00	1.10
2.10	1.00
2.20	1.00
2.30	1.00

11 Free energy surface with Labeled Simulation Snapshots

Figure S10 shows the liquid phase PMF with labels for different configurations along the profile. Reactive complex trimer geometries are shown in Figures S11, S12 and S13 for the liquid, gas phase and ionic liquid/vapor interface. Note that the liquid and gas phase configurations are noticeably different from one another past point a) of the profile, while the liquid and liquid/vapor interface configurations are fairly similar. For example, in Figure S13c the nonreactive spectator acetate for Reaction 1 in the reactive complex is observed to have a similar proximity to the $EMIM^+$ ethyl group as we see in the liquid phase simulations Figure S11c. This acetate was positioned over the imidazolium ring in the gas phase, as can be seen in Figure S12c.

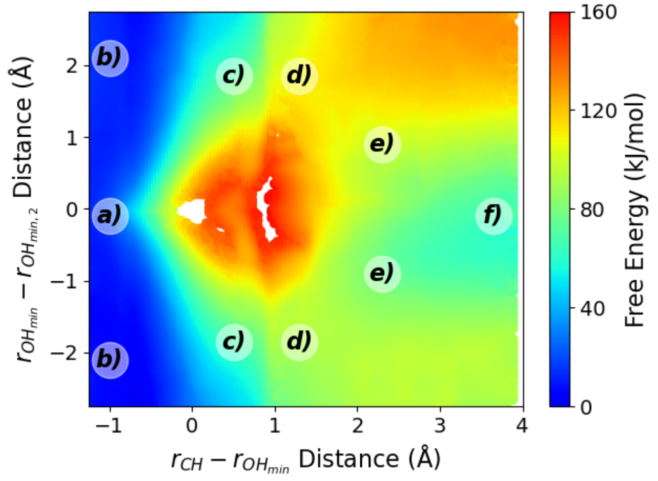

Figure S10: Free energy surface of reaction 1 and 2 in the liquid phase. The labels correspond to individual panels in Figures S11-13.

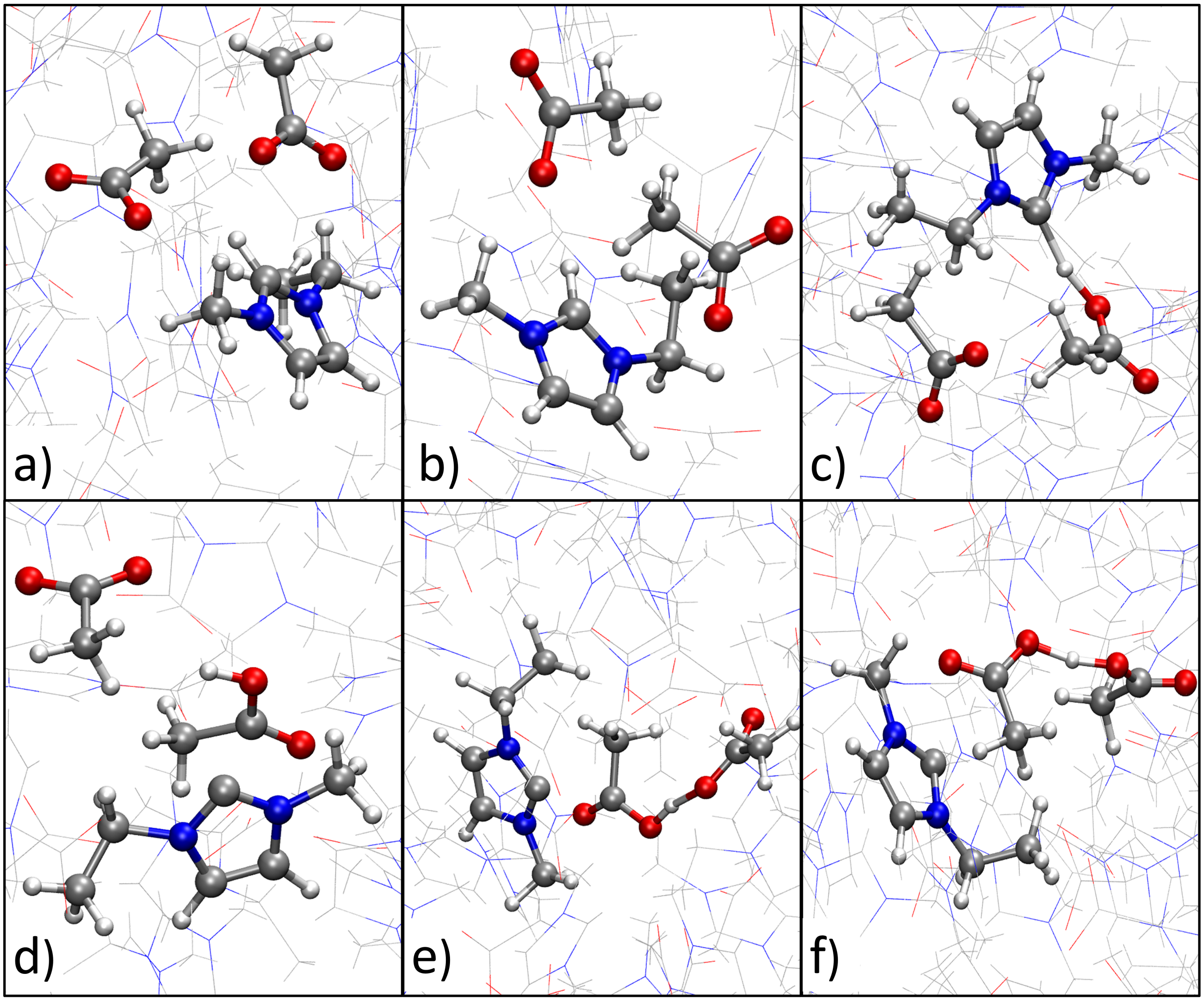

Figure S11: Simulation frames showing proton transfer from $EMIM^+$ to the $AcOH/OAc^-$ dimer in the bulk liquid. The location of each panel a-f on the reaction profile can be seen in Figure S10.

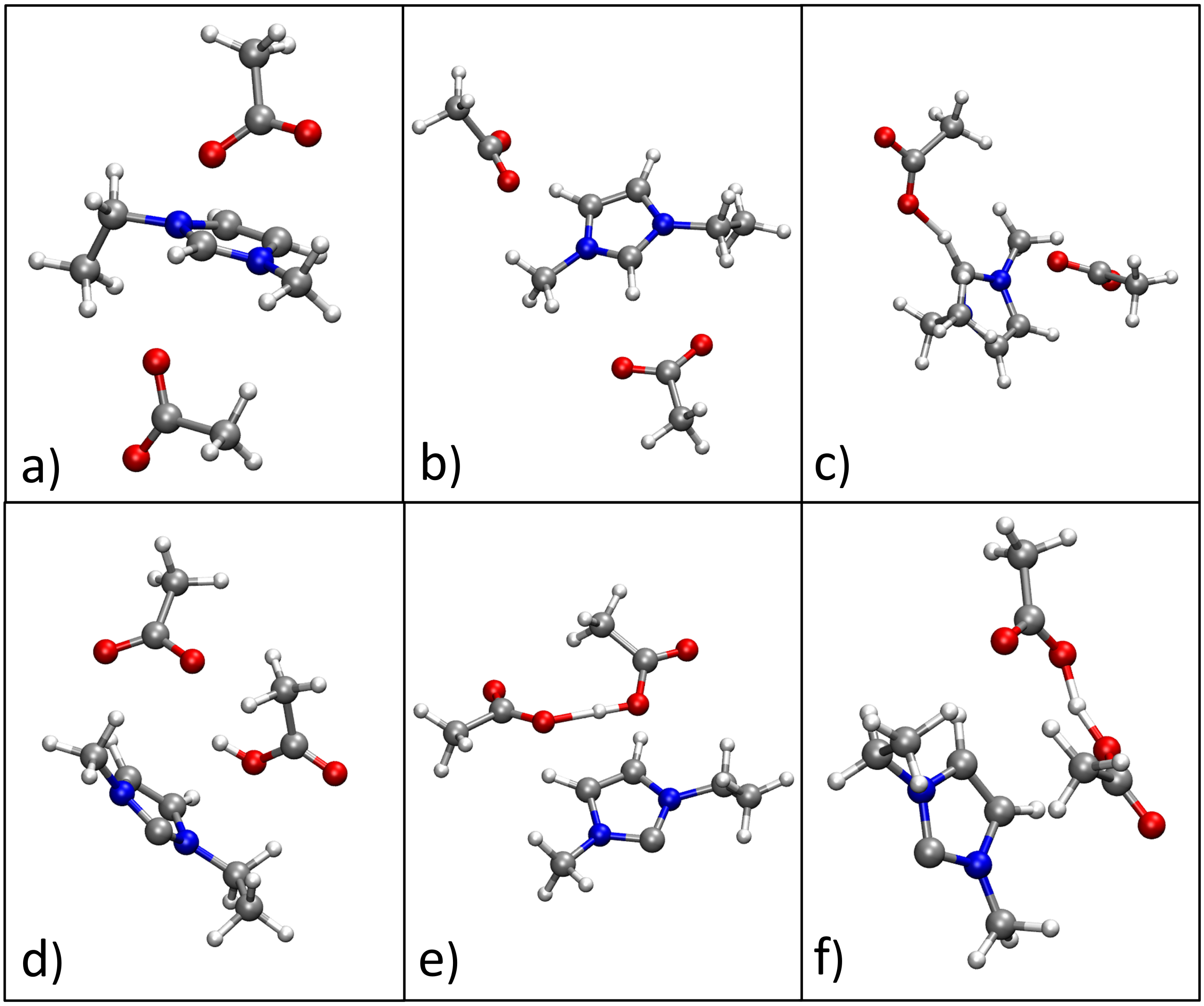

Figure S12: Simulation frames showing proton transfer from $EMIM^+$ to the $AcOH/OAc^-$ dimer in the gas phase. The location of each panel a-f on the reaction profile is the same as in the liquid phase and can be seen in Figure S10.

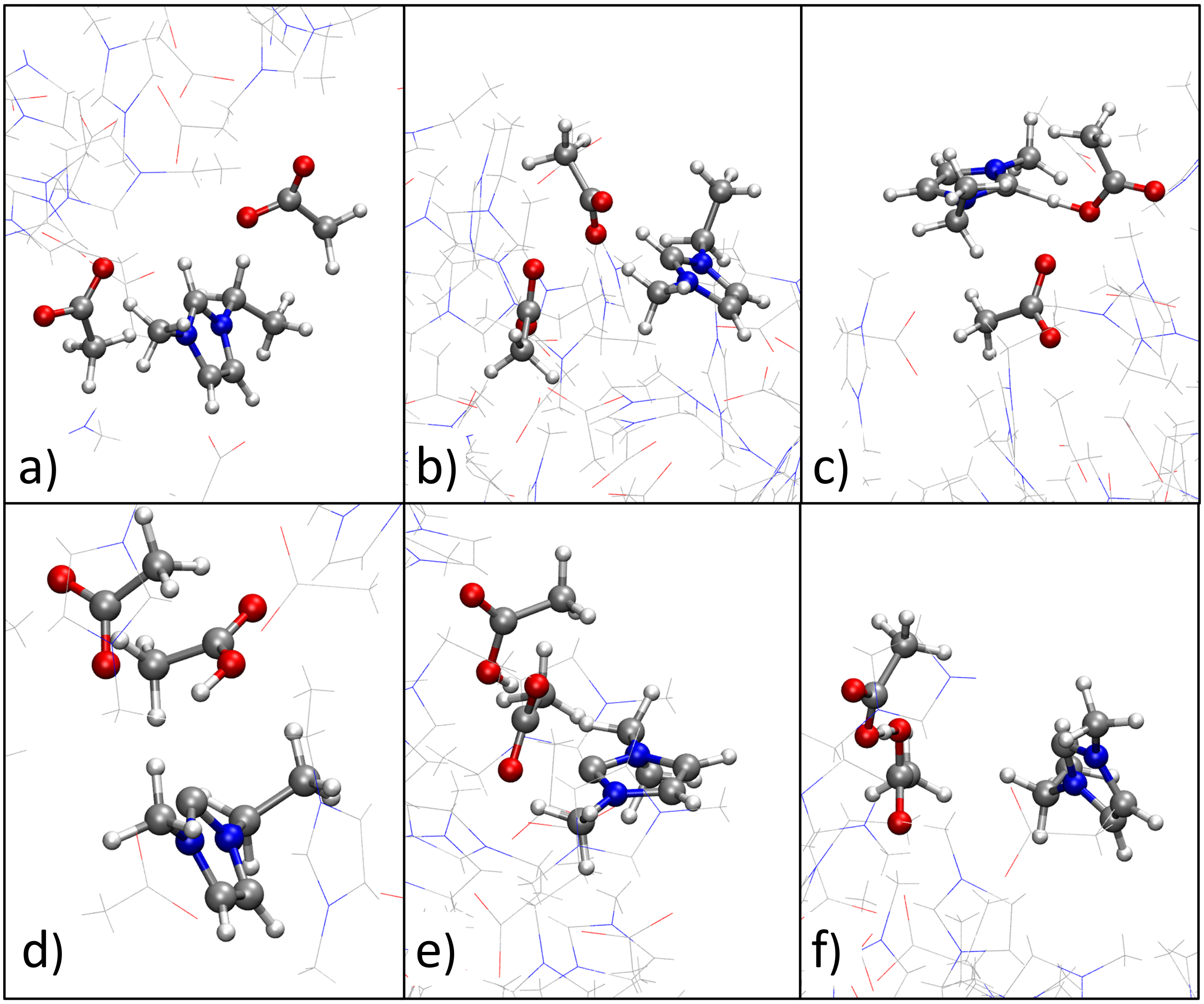

Figure S13: Simulation frames showing proton transfer from $EMIM^+$ to the AcOH/OAc^- dimer at the liquid/vapor interface. The location of each panel a-f on the reaction profile is the same as in the liquid phase and can be seen in Figure S10.

12 NHC ring center of mass / AcOH - OAc^- Dimer center of mass distance

The histograms in the left column of Figure S14 are of the distance between the NHC center of mass and the AcOH/OAc^- dimer center of mass from the liquid and gas phase. The histograms in the right column are of the angle between the C2 carbon, the NHC center of mass and the AcOH/OAc^- dimer center of mass. The collective variable values that each histogram was constructed from (with the collective variables defined in Equations 5a and 5b of the Main Text) are listed in each figure title. Figure S15 shows the same plots but for the liquid phase and liquid/vapor interface. The histograms are noticeably different for the gas and liquid phase, while they are similar for the liquid phase and the liquid/vapor interface. Note that the angle distributions are broader in both the gas and liquid/vapor interface than in the liquid phase. This due to the absence of solvating ions in the gas phase and lower ion density at the interface.

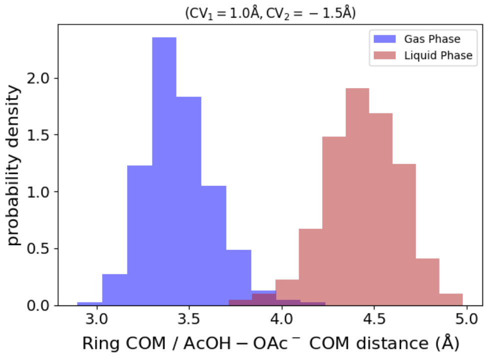

($CV_1 = 1.0Å, CV_2 = -1.5Å$)
Gas Phase
Liquid Phase
probability density
Ring COM / AcOH − OAc⁻ COM distance (Å)

(a)

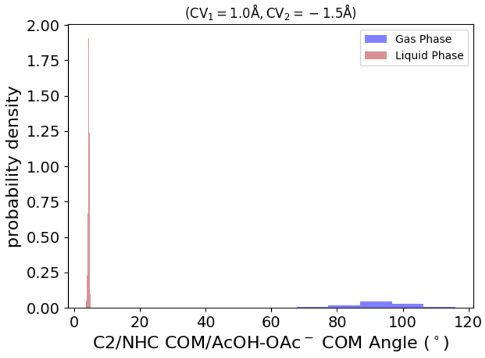

($CV_1 = 1.0Å, CV_2 = -1.5Å$)
Gas Phase
Liquid Phase
probability density
C2/NHC COM/AcOH-OAc⁻ COM Angle (°)

(b)

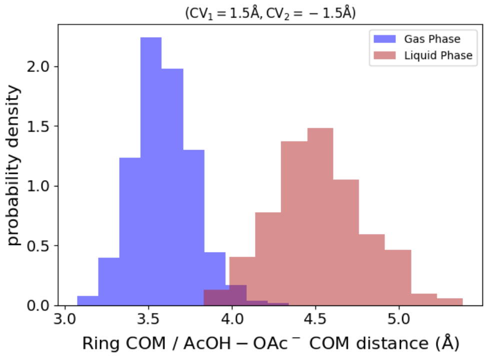

($CV_1 = 1.5Å, CV_2 = -1.5Å$)
Gas Phase
Liquid Phase
probability density
Ring COM / AcOH − OAc⁻ COM distance (Å)

(c)

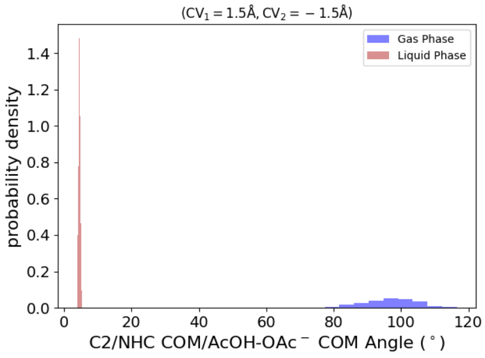

($CV_1 = 1.5Å, CV_2 = -1.5Å$)
Gas Phase
Liquid Phase
probability density
C2/NHC COM/AcOH-OAc⁻ COM Angle (°)

(d)

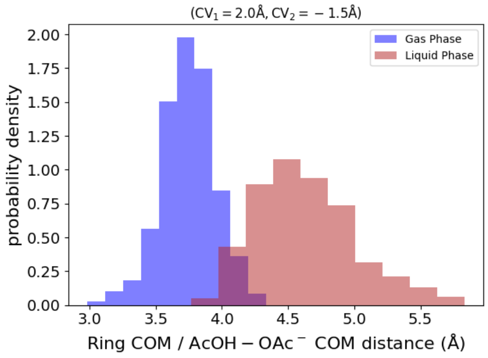

($CV_1 = 2.0Å, CV_2 = -1.5Å$)
Gas Phase
Liquid Phase
probability density
Ring COM / AcOH − OAc⁻ COM distance (Å)

(e)

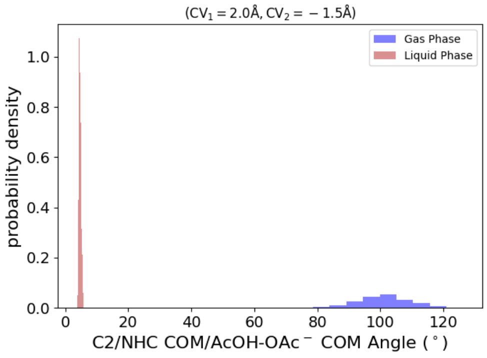

($CV_1 = 2.0Å, CV_2 = -1.5Å$)
Gas Phase
Liquid Phase
probability density
C2/NHC COM/AcOH-OAc⁻ COM Angle (°)

(f)

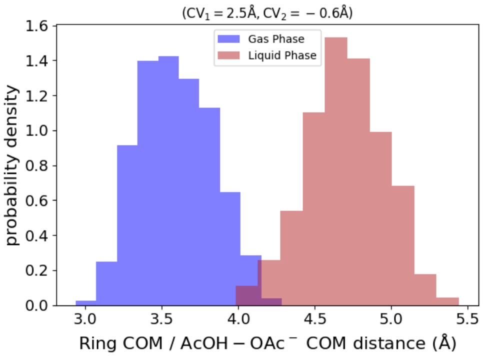

(g)

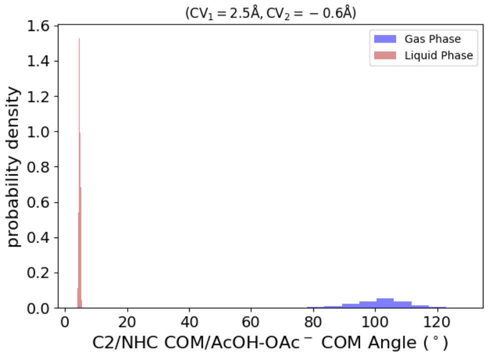

(h)

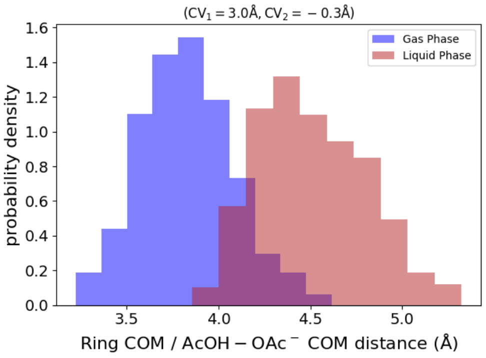

(i)

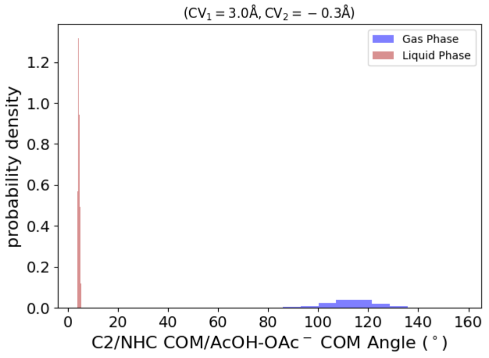

(j)

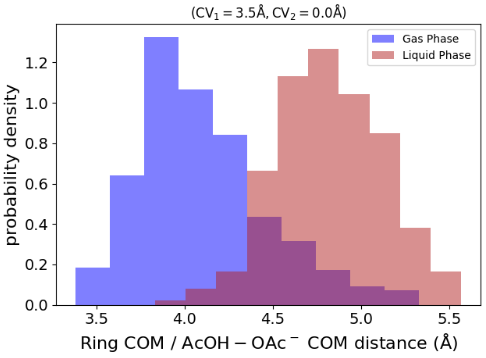

(k)

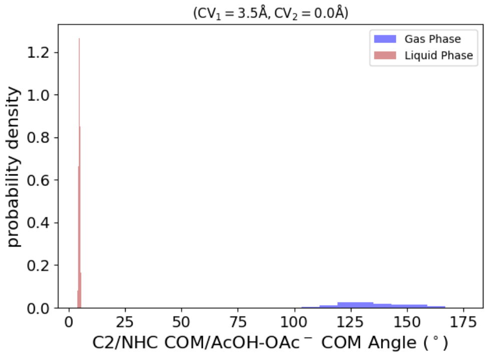

(l)

Figure S14: Each row contains two histograms per window. The first column is a histogram of the NHC center of mass and AcOH/OAc^- dimer center of mass distance. The second histogram in the row is a histogram of the angle between the C2 carbon, the NHC center of mass and the AcOH/OAc^- center of mass. Results are shown for the liquid and gas phase.

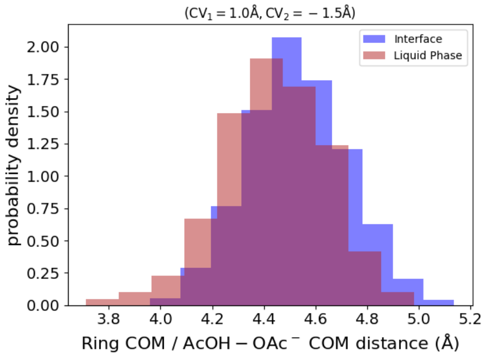
(CV$_1$ = 1.0Å, CV$_2$ = − 1.5Å)
Interface
Liquid Phase
probability density
Ring COM / AcOH − OAc$^-$ COM distance (Å)

(a)

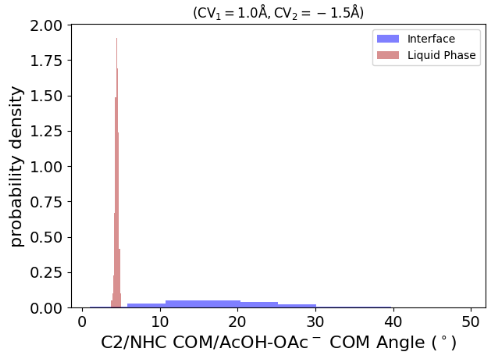
(CV$_1$ = 1.0Å, CV$_2$ = − 1.5Å)
Interface
Liquid Phase
probability density
C2/NHC COM/AcOH-OAc$^-$ COM Angle (°)

(b)

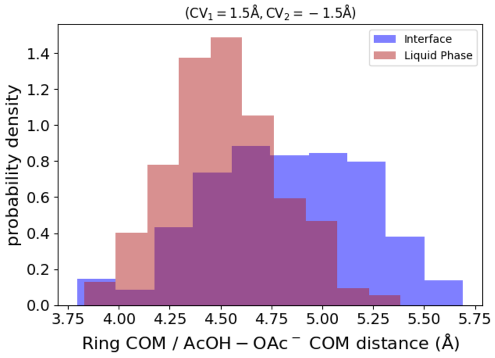
(CV$_1$ = 1.5Å, CV$_2$ = − 1.5Å)
Interface
Liquid Phase
probability density
Ring COM / AcOH − OAc$^-$ COM distance (Å)

(c)

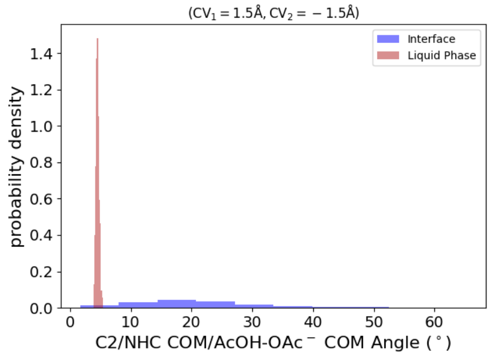
(CV$_1$ = 1.5Å, CV$_2$ = − 1.5Å)
Interface
Liquid Phase
probability density
C2/NHC COM/AcOH-OAc$^-$ COM Angle (°)

(d)

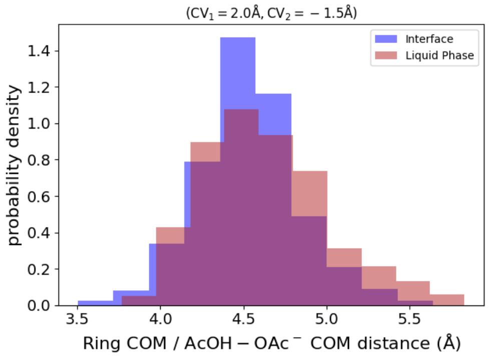
(CV$_1$ = 2.0Å, CV$_2$ = − 1.5Å)
Interface
Liquid Phase
probability density
Ring COM / AcOH − OAc$^-$ COM distance (Å)

(e)

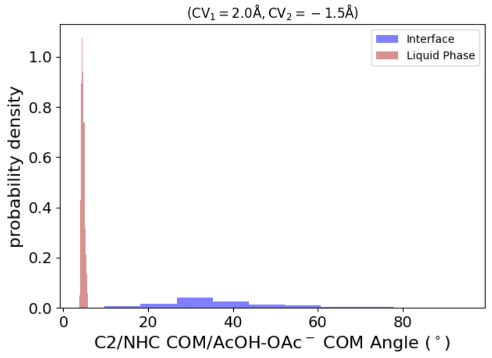
(CV$_1$ = 2.0Å, CV$_2$ = − 1.5Å)
Interface
Liquid Phase
probability density
C2/NHC COM/AcOH-OAc$^-$ COM Angle (°)

(f)

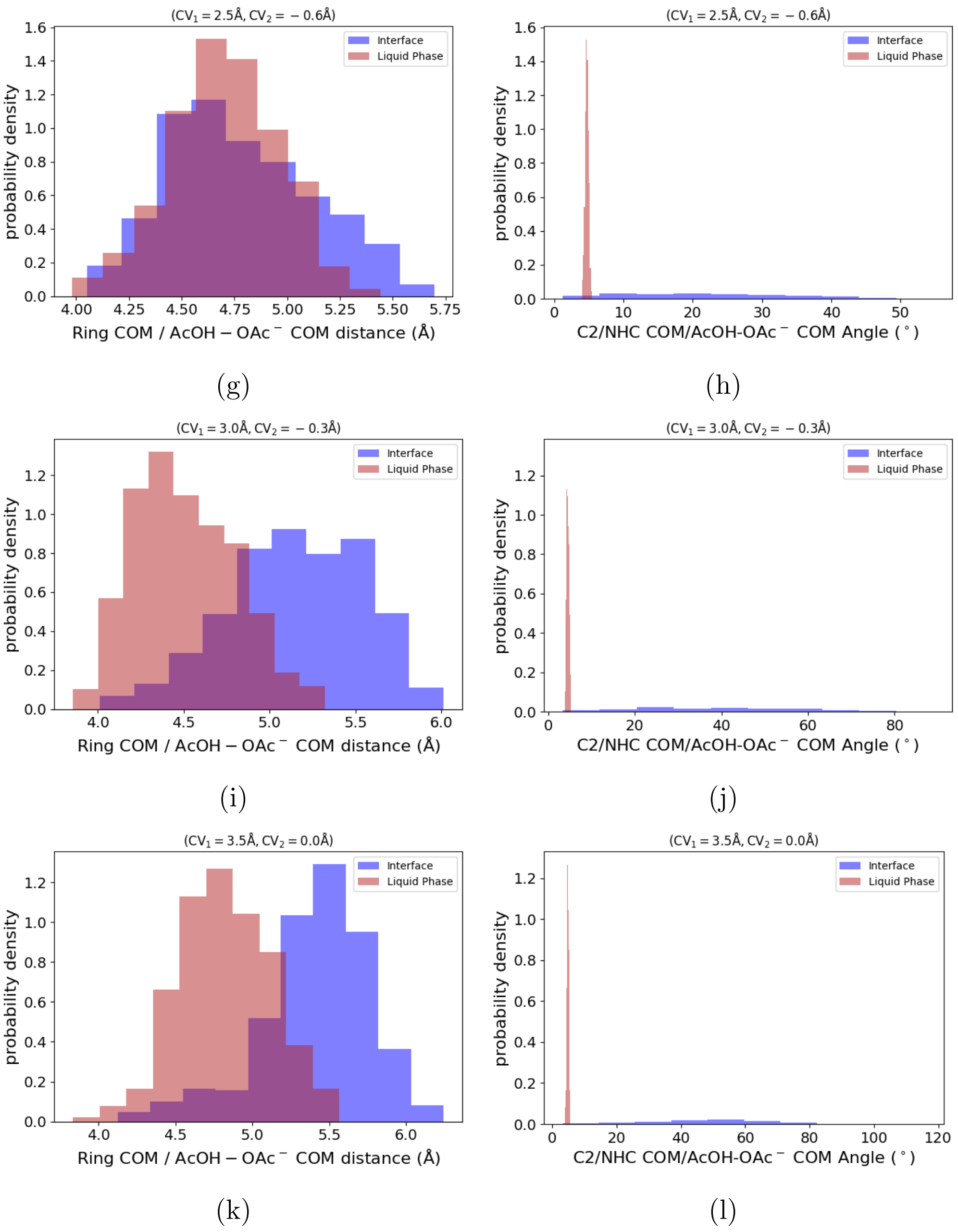

Figure S15: Each row contains two histograms per window. The first column is a histogram of the NHC center of mass and AcOH/OAc^- dimer center of mass distance. The second histogram in each row is a histogram of the angle between the C2 carbon, the NHC center of mass and the AcOH/OAc^- center of mass. Results are shown for the liquid and liquid/vapor interface.

13 Spatial Distribution Functions

We plot spatial distribution functions of the $EMIM^+$ ring surrounded by oxygen atoms for the collective variable values of ($CV_1 = -1.0$ Å, $CV_2 = \pm 2.0$ Å); the SDFs were constructed using Travis.[S15] The different configurations configurations for these two windows leads to the asymmetry in the liquid PMF.

We also show SDFs from the liquid and liquid/vapor interface in Figure 11 of the Main Text, with both corresponding to the R_1 configuration. Except for the nonreactive ring proton near the ethyl group, the oxygen density is similar.

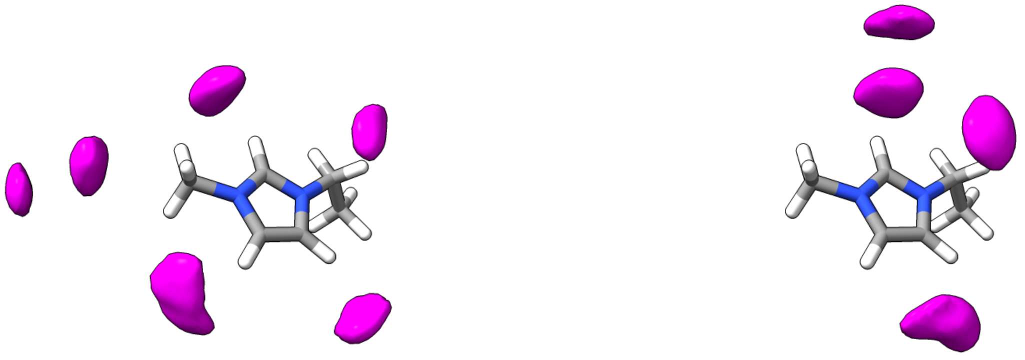

(a) (b)

Figure S16: SDFs of the $EMIM^+$ ring for a) a liquid phase simulation with collective variable values ($CV_1 = -1.0$ Å, $CV_2 = -2.0$ Å) and b) a liquid phase simulation with collective variable values ($CV_1 = -1.0$ Å, $CV_2 = 2.0$ Å). The isovalue was set equal to 1.38 $nm^{-}3$.

14 EMIM$^+$ H3 and H4 ring protons/oxygen RDF

Figure S17 shows ρg(r) between the (reacting complex) EMIM$^+$ nonreactive H3 and H4 protons and the acetate oxygens for 3 different umbrella sampling windows in the bulk liquid. The window constraints are listed in the Figure S17 caption.

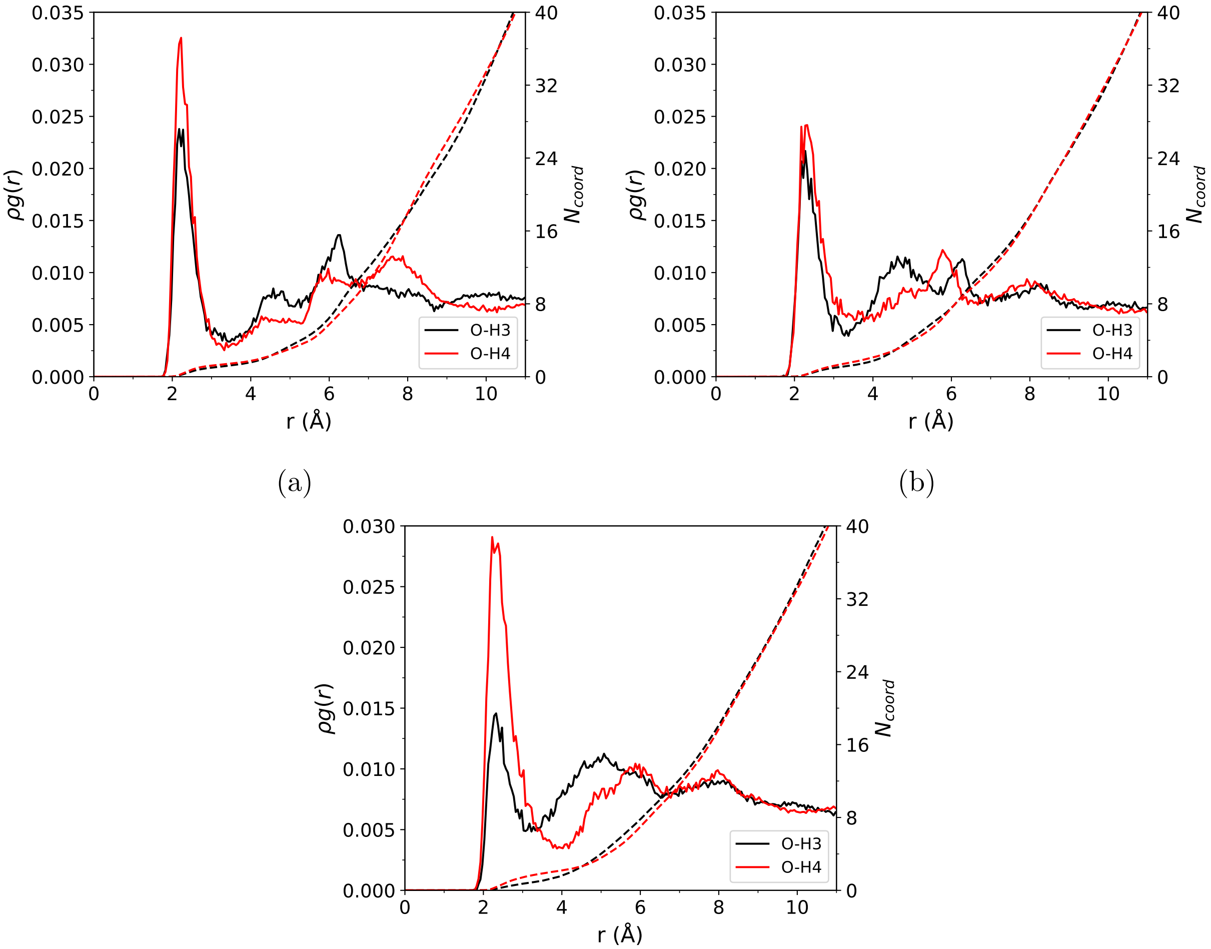

(a) (b)

Figure S17: $\rho g(r)$ for the reactive EMIM$^+$ H3/acetate oxygen atoms and $\rho g(r)$ for the reactive EMIM$^+$ H4/ acetate oxygen atoms. The windows the RDFs are computed from are a)($CV_1 = -1.0$ Å, $CV_2 = -2.0$ Å) b) ($CV_1 = -1.0$ Å, $CV_2 = 2.0$ Å) c) ($CV_1 = -1.0$ Å, $CV_2 = 0.0$ Å)

15 Ring proton - oxygen RDFs

In Figure S18, we show the combined H3/H4 ring proton/oxygen atom $\rho g(r)$ distributions for both the "Reactant" $EMIM^+$ (R_1) and "Product" NHC (P_2) states mentioned in the Main Text. The hydrogen bond between these molecules is much stronger for the Reactant. In Figure S19, we show the Reactant $EMIM^+$ and Product NHC $\rho g(r)$ decomposed between H3 and H4. Figure S19b shows that there is small acetate presence at the H3 proton and negligible presence at the H4 proton for the NHC. These simulations are taken from the liquid phase.

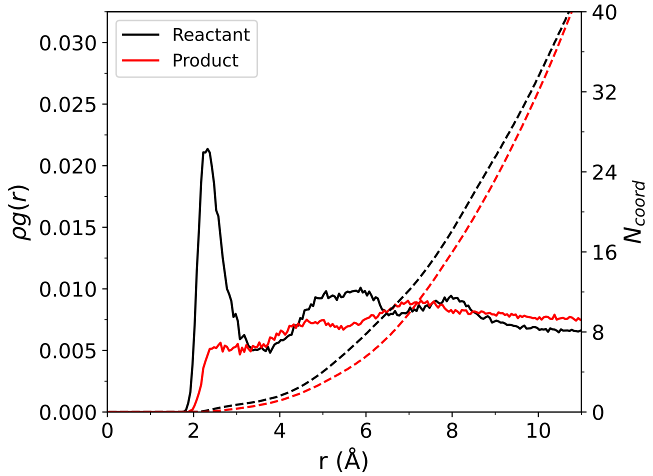

Figure S18: $\rho g(r)$ for the reactive $EMIM^+$ H3 and H4 proton/acetate oxygen atoms and $\rho g(r)$ for the NHC H3 and H4 protons/oxygens. The "Reactant" simulation is from a window with ($CV_1 = -1.0$ Å, $CV_2 = 0.0$ Å) and the "Product" simulation is from a window with ($CV_1 = 3.5$ Å, $CV_2 = 0.0$ Å).

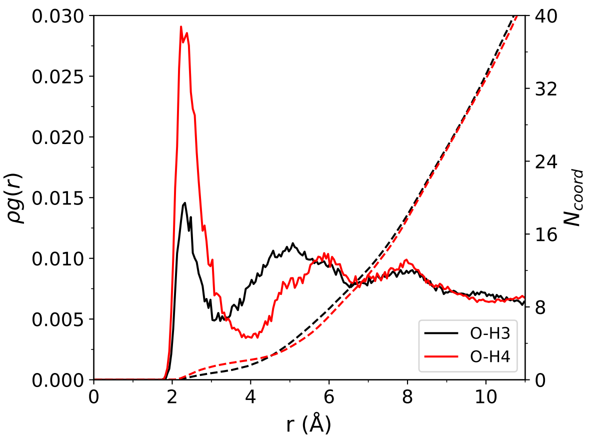

(a)

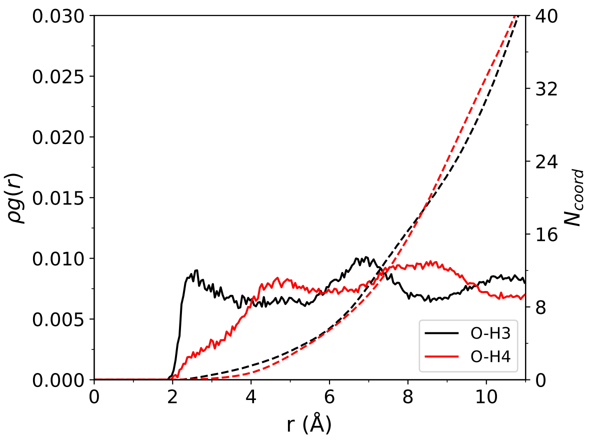

(b)

Figure S19: a) RDF of the reactive complex $EMIM^+$ H3 and H4 protons with solvent acetate oxygen atoms. This is a replot of Figure S17c. b) RDF of the reactive complex NHC product H3 and H4 protons with solvent acetate oxygen atoms.

16 Reacting Complex Atom Labeling

We follow the atom labeling convention in the reactive complex for some sections of the paper. The reactive complex is considered to be composed of $EMIM^+$ and two acetates, and allows for solvent acetates to switch in and out of the reactive complex.

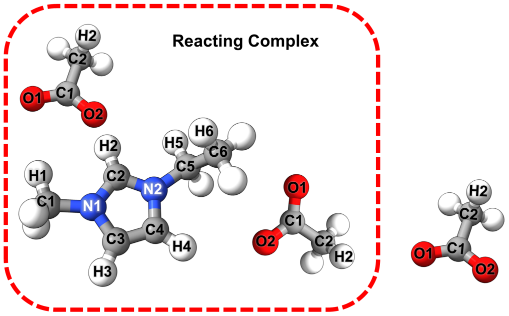

Figure S20: Reactive complex and atom naming convention in this work.

17 NHC Liquid/Vapor Interface PMF

In order to assess the relative stability of the NHC at the liquid/vapor interface in comparison to the bulk liquid, we performed nonreactive umbrella sampling simulations of the NHC in the liquid/vapor interface system. The umbrella sampling simulations are of a system consisting of the NHC, acetic acid and 39 [$EMIM^+$][OAc^-] ion pairs, and are nonreactive; this prevents simulation of the AcOH/OAc^- dimer, but allows for focusing on the environmental preference of the NHC. The z-coordinate of the NHC center of mass was biased from the center of mass of the simulation box (set at the origin, 0.0 Å) to 20 Å in 0.5 Å increments. A 20 kJ/mol force constant was used for the harmonic umbrella potentials. The simulation settings are the same as those mentioned in the main text. The 1D PMF is shown in Figure S21. The minimum free energy region is at $\sim$ 11 Å, which is approximately the location of the Gibbs dividing surface. There is a 15-20 kJ/mol barrier for the NHC to move further into the liquid or into the vacuum.

From our results, it appears that there is a positive solvation energy for the NHC in the IL. However, it has been found experimentally that aromatic compounds such as benzene are solvated to a limited extent in [$EMIM^+$][OAc^-], which implies a small solvation energy for the NHC here.[S16] Determining the solvation energy of NHC within statistical accuracy would require more extensive simulation, which is beyond the scope of this work.

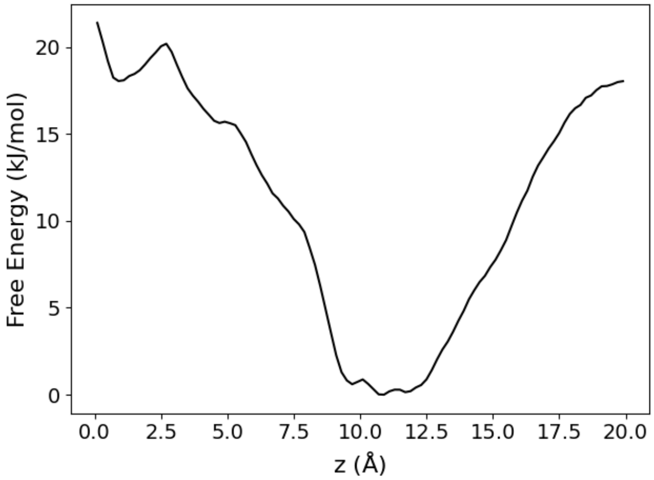

Figure S21: Potential of mean force for the NHC center of mass along the z-dimension of the simulation box.

18 Estimate of NHC Concentration in the Bulk Liquid

Here we give an order of magnitude estimate for the NHC concentration based off of our free energy results for the liquid and liquid-interface systems. The equilibrium coefficient is given by the ratio of the forward and reverse rate constants for the reaction:

$$K = \frac{k_f}{k_r} \tag{S11}$$

Considering the reaction:

$$EMIM^+ + OAc^- \longleftrightarrow NHC + AcOH \tag{S12}$$

the forward and reverse rates are given through second-order kinetics as:

$$v_f^{eq} = k_f[EMIM^+][OAc^-] \tag{S13}$$

$$v_r^{eq} = k_r[NHC][AcOH] \tag{S14}$$

with the forward and reverse rates equal to one another at equilibrium. Additionally, the

rate constants can be related to the free energy through the well-known expression:

$$k_f = A_f e^{-\beta \Delta G_f^\ddagger} \tag{S15}$$

$$k_r = A_r e^{-\beta \Delta G_r^\ddagger} \tag{S16}$$

The ratio of the forward and reverse rate constants allows for the relation of the concentrations of the species to the free energy:

$$\frac{k_f}{k_r} = \frac{[NHC][AcOH]}{[EMIM^+][OAc^-]} \approx e^{-\beta \Delta G} \tag{S17}$$

where we have assumed that the prefactors in Equation S15 and S16 are approximately equal to one another. The concentration of $EMIM^+$ and OAc^- in the bulk IL is equal to 6.1 M. Entering this in to Equation S17 and equating the concentration of NHC with that of acetic acid gives:

$$[NHC] \approx (e^{-70/2.5} 6.1^2)^{1/2} = 5.1\mu\text{M} \tag{S18}$$

in the liquid phase and

$$[NHC] \approx (e^{-60/2.5} 6.1^2)^{1/2} = 37.9\mu\text{M} \tag{S19}$$

for the liquid/vapor interface. Note that the free energies above are approximate and read off of Figure 9 in the main text. These concentrations should be treated as order of magnitude estimates rather than exact figures.

19 PB/NN Timings

The Hamiltonian in Equation 3 of the main text requires 3 calls to the diagonal terms in Equation 4, plus evaluation of the 3 off-diagonal elements. Of the terms in Equation 4, only the force field calls ($E^{Bonded,FF}_{Solvent}$ and $E^{Nonbonded,FF}_{Solute,Solvent}$) scale with respect to the number of solvent molecules in the system. The other terms (E^{Morse}_{Solute}, $E^{Intra,NN}_{Solute}$ and $E^{Inter,NN}_{Solute}$) are only dependent on the reactive complex and will not scale with system size as long as the reactive complex stays fixed. The H_{ij} terms are similarly only dependent on the reactive complex. The average timings for various operations performed on a NVIDIA TITAN Xp GPU can be seen in Table S6. For the diagonal terms, note that each timing is the average time each operation takes per "state" of the Hamiltonian. The H_{12} timing only includes the average time for this specific off-diagonal element of the Hamiltonian. The collection of force field terms ($E^{Bonded,FF}_{Solvent} + E^{Nonbonded,FF}_{Solute,Solvent} + E^{Morse}_{Solute}$) is the slowest part of the simulation and will scale with respect to the size of the system. This is due to various inefficiencies in the current code implementation associated with sending the full system positions and forces back and forth from the CPU and GPU.

A 1 ps simulation of the bulk liquid system described in the Main Text using the PB/NN Hamiltonian takes 833.7 seconds to complete. For comparison, we also ran a 1 ps simulation of a nonreactive polarizable force field simulation of 40 [$EMIM^+$][OAc^-] pairs (essentially $E^{Bonded,FF}_{Solvent} + E^{Nonbonded,FF}_{Solute,Solvent}$). OpenMM was used for this simulation.[S17] The Drude SCF Integrator was used for this system with a timestep of 0.5 fs; an anharmonic restraining potential was used for the Drude oscillators.[S18] The OpenCL OpenMM kernels with mixed precision were used for this simulation. Other simulation settings are the same as those used for the reactive simulations (mentioned in the Methods section of the Main Text). This simulation ran for 141.9 seconds, which is 5.88 times faster than the reactive PB/NN simulation. A code requiring a lower extent of CPU/GPU communication would reduce the differential between the two simulations, and we will pursue this in future work. For comparison, we also ran simulations with the nonreactive polarizable force field using the Drude Langevin integrator,

also run using the OpenCL kernels in OpenMM.[S18] This simulation was approximately 180 times faster than the PB/NN simulation. Note that the PB/NN procedure uses the Drude SCF method to calculate the polarization energy, which is less computationally efficient than the extended Lagrangian method used in the Drude Langevin integrator.

Table S6: Timings for various PB/NN operations.

Operation	Time (s)
$E_{Solvent}^{Bonded,FF} + E_{Solute,Solvent}^{Nonbonded,FF} + E_{Solute}^{Morse}$	0.048
$E_{Solute}^{Intra,NN}$	0.042
$E_{Solute}^{Inter,NN}$	0.029
H_{ij}	0.0079

References

(S1) Kühne, T. D.; Iannuzzi, M.; Del Ben, M.; Rybkin, V. V.; Seewald, P.; Stein, F.; Laino, T.; Khaliullin, R. Z.; Schütt, O.; Schiffmann, F.; Golze, D.; Wilhelm, J.; Chulkov, S.; Bani-Hashemian, M. H.; Weber, V.; Borštnik, U.; Taillefumier, M.; Jakobovits, A. S.; Lazzaro, A.; Pabst, H.; Müller, T.; Schade, R.; Guidon, M.; Andermatt, S.; Holmberg, N.; Schenter, G. K.; Hehn, A.; Bussy, A.; Belleflamme, F.; Tabacchi, G.; Glöß, A.; Lass, M.; Bethune, I.; Mundy, C. J.; Plessl, C.; Watkins, M.; VandeVondele, J.; Krack, M.; Hutter, J. CP2K: An electronic structure and molecular dynamics software package - Quickstep: Efficient and accurate electronic structure calculations. *The Journal of Chemical Physics* **2020**, *152*, 194103.

(S2) Perdew, J. P.; Burke, K.; Ernzerhof, M. Generalized gradient approximation made simple. *Physical Review Letters* **1996**, *77*, 3865–3868.

(S3) Goedecker, S.; Teter, M. Separable dual-space Gaussian pseudopotentials. *Physical Review B - Condensed Matter and Materials Physics* **1996**, *54*, 1703–1710.

(S4) Grimme, S.; Antony, J.; Ehrlich, S.; Krieg, H. A consistent and accurate ab initio parametrization of density functional dispersion correction (DFT-D) for the 94 elements H-Pu. *Journal of Chemical Physics* **2010**, *132*, 154104.

(S5) Smith, D. G.; Burns, L. A.; Simmonett, A. C.; Parrish, R. M.; Schieber, M. C.; Galvelis, R.; Kraus, P.; Kruse, H.; Di Remigio, R.; Alenaizan, A.; James, A. M.; Lehtola, S.; Misiewicz, J. P.; Scheurer, M.; Shaw, R. A.; Schriber, J. B.; Xie, Y.; Glick, Z. L.; Sirianni, D. A.; O'Brien, J. S.; Waldrop, J. M.; Kumar, A.; Hohenstein, E. G.; Pritchard, B. P.; Brooks, B. R.; Schaefer, H. F.; Sokolov, A. Y.; Patkowski, K.; DePrince, A. E.; Bozkaya, U.; King, R. A.; Evangelista, F. A.; Turney, J. M.; Crawford, T. D.; Sherrill, C. D. Psi4 1.4: Open-source software for high-throughput quantum chemistry. *The Journal of chemical physics* **2020**, *152*, 184108.

(S6) Stoppelman, J. P.; McDaniel, J. G. Physics-based, neural network force fields for reactive molecular dynamics: Investigation of carbene formation from [EMIM$^+$][OAc$^-$]. *J. Chem. Phys.* **2021**, *155*, 104112.

(S7) Glick, Z. L.; Metcalf, D. P.; Koutsoukas, A.; Spronk, S. A.; Cheney, D. L.; Sherrill, C. D. AP-Net: An atomic-pairwise neural network for smooth and transferable interaction potentials. *J. Chem. Phys.* **2020**, *153*, 044112.

(S8) Behler, J. Neural network potential-energy surfaces in chemistry: A tool for large-scale simulations. **2011**, *13*, 17930–17955.

(S9) Schütt, K.; Kindermans, P.-J.; Felix, H. E. S.; Chmiela, S.; Tkatchenko, A.; Müller, K.-R. SchNet: A continuous-filter convolutional neural network for modeling quantum interactions. 2017; http://papers.nips.cc/paper/6700-schnet-a-continuous-filter-convolutional-neural-network-for-modeling-quantum-interactions.

(S10) Kingma, D. P.; Ba, J. L. Adam: A method for stochastic optimization. 3rd International Conference on Learning Representations, ICLR 2015 - Conference Track Proceedings. 2015.

(S11) Lim, V. T.; Bayly, C. I.; Fusti-Molnar, L.; Mobley, D. L. Assessing the Conformational Equilibrium of Carboxylic Acid via Quantum Mechanical and Molecular Dynamics Studies on Acetic Acid. *Journal of Chemical Information and Modeling* **2019**, *59*, 1957–1964.

(S12) Hjorth Larsen, A.; JØrgen Mortensen, J.; Blomqvist, J.; Castelli, I. E.; Christensen, R.; Dułak, M.; Friis, J.; Groves, M. N.; Hammer, B.; Hargus, C.; Hermes, E. D.; Jennings, P. C.; Bjerre Jensen, P.; Kermode, J.; Kitchin, J. R.; Leonhard Kolsbjerg, E.; Kubal, J.; Kaasbjerg, K.; Lysgaard, S.; Bergmann Maronsson, J.; Maxson, T.; Olsen, T.; Pastewka, L.; Peterson, A.; Rostgaard, C.; SchiØtz, J.; Schütt, O.;

Strange, M.; Thygesen, K. S.; Vegge, T.; Vilhelmsen, L.; Walter, M.; Zeng, Z.; Jacobsen, K. W. The atomic simulation environment - A Python library for working with atoms. 2017; https://doi.org/10.1088/1361-648X/aa680e.

(S13) Bonomi, M.; Bussi, G.; Camilloni, C.; Tribello, G. A.; Banáš, P.; Barducci, A.; Bernetti, M.; Bolhuis, P. G.; Bottaro, S.; Branduardi, D.; Capelli, R.; Carloni, P.; Ceriotti, M.; Cesari, A.; Chen, H.; Chen, W.; Colizzi, F.; De, S.; De La Pierre, M.; Donadio, D.; Drobot, V.; Ensing, B.; Ferguson, A. L.; Filizola, M.; Fraser, J. S.; Fu, H.; Gasparotto, P.; Gervasio, F. L.; Giberti, F.; Gil-Ley, A.; Giorgino, T.; Heller, G. T.; Hocky, G. M.; Iannuzzi, M.; Invernizzi, M.; Jelfs, K. E.; Jussupow, A.; Kirilin, E.; Laio, A.; Limongelli, V.; Lindorff-Larsen, K.; Löhr, T.; Marinelli, F.; Martin-Samos, L.; Masetti, M.; Meyer, R.; Michaelides, A.; Molteni, C.; Morishita, T.; Nava, M.; Paissoni, C.; Papaleo, E.; Parrinello, M.; Pfaendtner, J.; Piaggi, P.; Piccini, G. M.; Pietropaolo, A.; Pietrucci, F.; Pipolo, S.; Provasi, D.; Quigley, D.; Raiteri, P.; Raniolo, S.; Rydzewski, J.; Salvalaglio, M.; Sosso, G. C.; Spiwok, V.; Šponer, J.; Swenson, D. W.; Tiwary, P.; Valsson, O.; Vendruscolo, M.; Voth, G. A.; White, A. Promoting transparency and reproducibility in enhanced molecular simulations. 2019.

(S14) Wang, L. P.; Song, C. Geometry optimization made simple with translation and rotation coordinates. *Journal of Chemical Physics* **2016**, *144*, 214108.

(S15) Brehm, M.; Thomas, M.; Gehrke, S.; Kirchner, B. TRAVIS—A free analyzer for trajectories from molecular simulation. *J. Chem. Phys.* **2020**, *152*, 164105.

(S16) Pereira, J. F.; Flores, L. A.; Wang, H.; Rogers, R. D. Benzene Solubility in Ionic Liquids: Working Toward an Understanding of Liquid Clathrate Formation. *Chemistry – A European Journal* **2014**, *20*, 15482–15492.

(S17) Eastman, P.; Swails, J.; Chodera, J. D.; McGibbon, R. T.; Zhao, Y.;

Beauchamp, K. A.; Wang, L. P.; Simmonett, A. C.; Harrigan, M. P.; Stern, C. D.; Wiewiora, R. P.; Brooks, B. R.; Pande, V. S. OpenMM 7: Rapid development of high performance algorithms for molecular dynamics. *PLOS Comput. Biol.* **2017**, *13*, e1005659.

(S18) Huang, J.; Lemkul, J. A.; Eastman, P. K.; MacKerell, A. D. Molecular dynamics simulations using the drude polarizable force field on GPUs with OpenMM: Implementation, validation, and benchmarks. *J. Comput. Chem.* **2018**, *39*, 1682–1689.
